# AI‐Driven TENGs for Self‐Powered Smart Sensors and Intelligent Devices

**DOI:** 10.1002/advs.202417414

**Published:** 2025-04-25

**Authors:** Aiswarya Baburaj, Syamini Jayadevan, Akshaya Kumar Aliyana, Naveen Kumar SK, George K Stylios

**Affiliations:** ^1^ Department of Electronics Mangalore University Mangalore 574199 India; ^2^ Research Institute for Flexible Materials School of Textiles and Design Heriot‐Watt University Netherdale Galashiels TD1 3HF United Kingdom of Great Britain and Northern Ireland

**Keywords:** artificial intelligence, deep learning, intelligent devices, machine learning, triboelectric nanogenerator

## Abstract

Triboelectric nanogenerators (TENGs) are emerging as transformative technologies for sustainable energy harvesting and precision sensing, offering eco‐friendly power generation from mechanical motion. They harness mechanical energy while enabling self‐sustaining sensing for self‐powered devices. However, challenges such as material optimization, fabrication techniques, design strategies, and output stability must be addressed to fully realize their practical potential. Artificial intelligence (AI), with its capabilities in advanced data analysis, pattern recognition, and adaptive responses, is revolutionizing fields like healthcare, industrial automation, and smart infrastructure. When integrated with TENGs, AI can overcome current limitations by enhancing output, stability, and adaptability. This review explores the synergistic potential of AI‐driven TENG systems, from optimizing materials and fabrication to embedding machine learning and deep learning algorithms for intelligent real‐time sensing. These advancements enable improved energy harvesting, predictive maintenance, and dynamic performance optimization, making TENGs more practical across industries. The review also identifies key challenges and future research directions, including the development of low‐power AI algorithms, sustainable materials, hybrid energy systems, and robust security protocols for AI‐enhanced TENG solutions.

## Graphical abstract

Triboelectric nanogenerators (TENGs) enable sustainable energy harvesting and self‐powered sensing but face challenges in material optimization, fabrication, and stability. Integrating artificial intelligence (AI) enhances TENG performance through machine learning, improving energy output, adaptability, and predictive maintenance. This review explores AI‐driven TENG advancements, key challenges, and future research directions for practical applications.

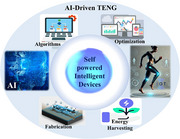

## 1 Introduction

The rapid advancements in artificial intelligence (AI) and triboelectric nanogenerators (TENGs) are paving the way for revolutionary self‐powered sensing technologies that promise to shape the future of intelligent, sustainable devices. AI's capabilities in data processing, pattern recognition, and predictive analysis have transformed sectors, such as healthcare, energy, finance, and manufacturing by enabling more efficient and adaptive systems.^[^ ^1^, ^2^, ^3^, ^4^ ^]^ Meanwhile, TENGs offer a sustainable solution for autonomous energy systems, efficiently converting mechanical energy from natural movements and environmental vibrations into electrical energy.^[^ ^5^, ^6^ ^]^ These devices provide a reliable alternative to conventional power sources, especially in remote or challenging environments.^[^ ^7^ ^]^ Their integration as self‐powered sensors offers advantages, such as low cost, high sensitivity, and stability, making them ideal for applications demanding energy independence, durability, and precision.^[^ ^8^, ^9^ ^]^ By combining AI and TENG technologies, a new generation of devices is emerging—capable of independent operation, continuous monitoring, real‐time data analysis, and dynamic adaptation to user‐specific needs. AI‐driven TENG systems optimize material selection, improve design strategies, and enhance application performance.^[^ ^10^, ^11^, ^12^ ^]^ Machine learning (ML) and deep learning (DL) techniques facilitate adaptive energy harvesting, predictive maintenance, and real‐time optimization, enabling more efficient and functional TENG systems.^[^ ^13^, ^14^ ^]^

**Figure 1:**
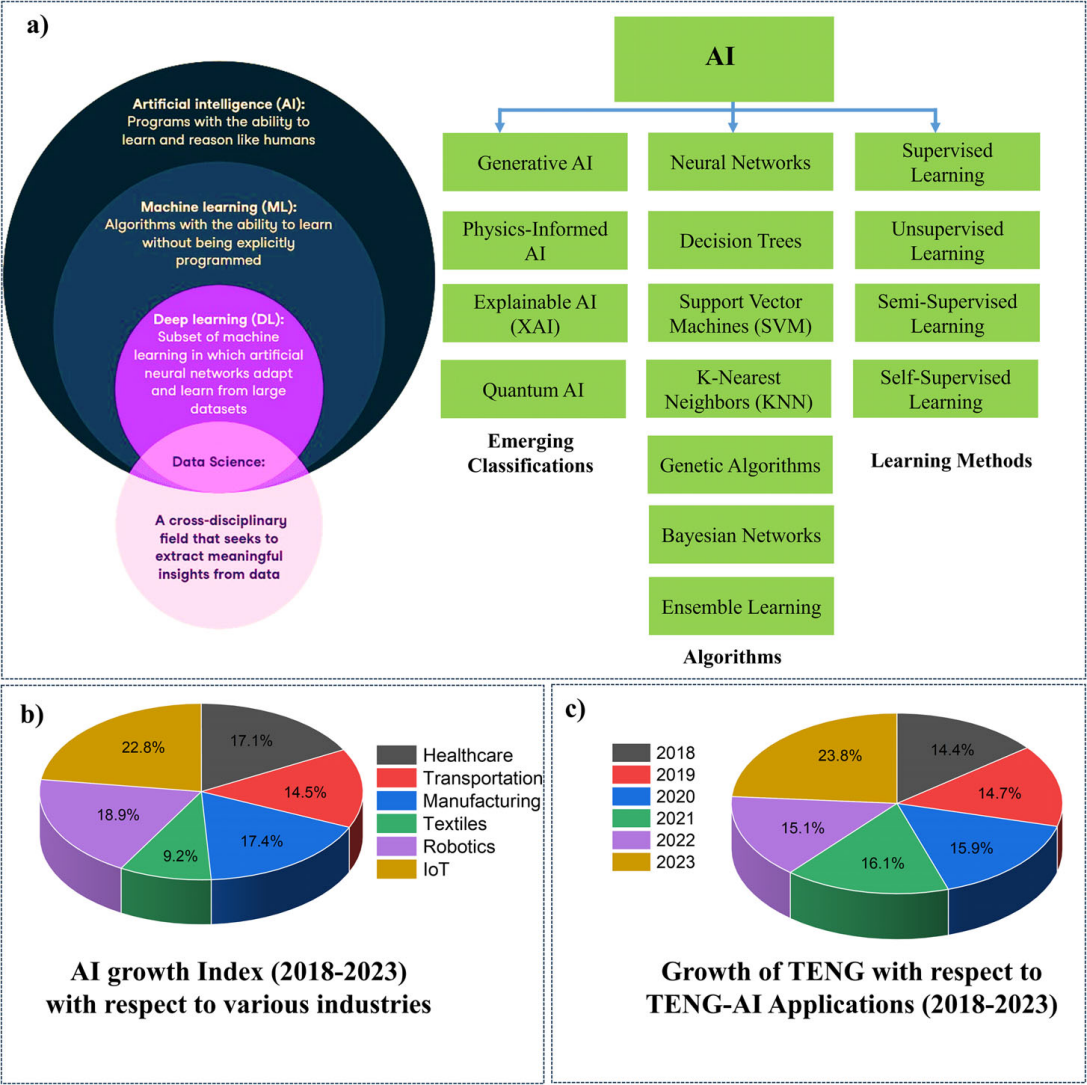
a) Overview of AI and its classifications. b) Illustrates the growth index of various industries from 2018 to 2023 and c) the growth of TENG papers and AI‐based TENG papers from 2018 to 2023. All essential copyrights and permissions received. (Using data from industry publications and bibliographic databases, we analyzed growth trends across various sectors, including the emerging field of AI‐powered TENG research (2018–2023)).

**Figure** **1** illustrates AI's classifications, highlighting foundational learning methods like Supervised, Unsupervised, and Self‐Supervised Learning, alongside advanced domains, such as Generative AI, Explainable AI, and Quantum AI. Key algorithms, including neural networks (NN), decision tree (DT), and support vector machine (SVM), are central to driving AI advancements across industries. These methodologies enhance TENG systems' capacity for autonomous adaptation, making them suitable for self‐powered sensor networks, biomedical devices, wearable electronics, and Internet of Things (IoT)‐enabled smart cities where energy independence and adaptability are essential. Figure 1b,c presents trends in industry adoption and research on AI‐enhanced TENG technologies from 2018 to 2023, showcasing the increasing demand for autonomous, energy‐efficient devices across sectors. The growing academic and industrial interest in AI‐powered TENGs underscores the importance of AI integration in unlocking TENG technology's full potential. Despite their promise, AI‐powered TENG systems face challenges, such as robust algorithm development, efficient power management, and seamless integration with sensor hardware. Addressing these challenges is critical to realizing the full potential of smart, self‐powered devices.

The integration of AI with TENGs has been widely explored in recent studies, each focusing on different aspects of AI‐TENG synergy.^[^ ^15^, ^16^, ^17^ ^]^ However, existing reviews tend to address specific applications rather than providing a complete, solution‐driven perspective. For instance, Zhou et al.^[^ ^18^ ^]^ discuss AI‐enhanced TENG applications in self‐powered sensors and intelligent systems but primarily focus on energy harvesting methods and ML applications without addressing broader system integration. Similarly, Jiao et al.,^[^ ^19^ ^]^ emphasize AI‐driven material selection and device optimization but lack a structured approach for fabrication, self‐repair mechanisms, and intelligent real‐time sensing. Duan et al.^[^ ^20^ ^]^ offer a detailed discussion on AI‐integrated triboelectric sensors, yet they do not establish a universal performance measurement framework for AI‐TENGs, limiting comparability and scalability​. Another major gap in the existing literature is energy efficiency and sustainability in AI‐TENG applications. While Zhou et al.^[^ ^18^ ^]^ and Xu et al.^[^ ^21^ ^]^ acknowledge the role of AI in enhancing energy harvesting, they do not propose specific strategies for developing low‐power AI models for real‐world deployment. In contrast, this review introduces TinyML, neuromorphic computing, and edge AI techniques, ensuring that AI‐TENG systems remain energy‐efficient and deployable in IoT‐based and wearable applications. Additionally, while Yu et al.^[^ ^22^ ^]^ explore TENGs in acoustic AI applications, they do not discuss scalability challenges in AI‐TENG integration within IoT and industrial automation. This review addresses this by outlining AI‐powered standardization techniques, ensuring that AI‐enhanced TENGs achieve consistent performance benchmarks and seamless integration into smart systems. The unique contribution of this review lies in its comprehensive AI‐TENG framework, which connects AI‐driven material discovery, fabrication techniques, energy optimization strategies, and large‐scale system applications. Unlike prior studies that treat these elements in isolation, we investigated a unified perspective that enhances scalability, efficiency, and real‐world applicability of AI‐TENGs. Moreover, we discussed AI‐based self‐adaptive control mechanisms, allowing AI‐TENG systems to dynamically optimize their performance based on environmental feedback and real‐time energy demands. Furthermore, this review presents a future‐ready roadmap for AI‐TENG evolution, highlighting emerging AI trends, such as federated learning, quantum AI, and bio‐inspired computing. These advanced AI methodologies will further enhance predictive modeling, adaptive learning, and energy efficiency in TENG systems, positioning them as key enablers for next‐generation self‐powered intelligent devices.

**Figure 2:**
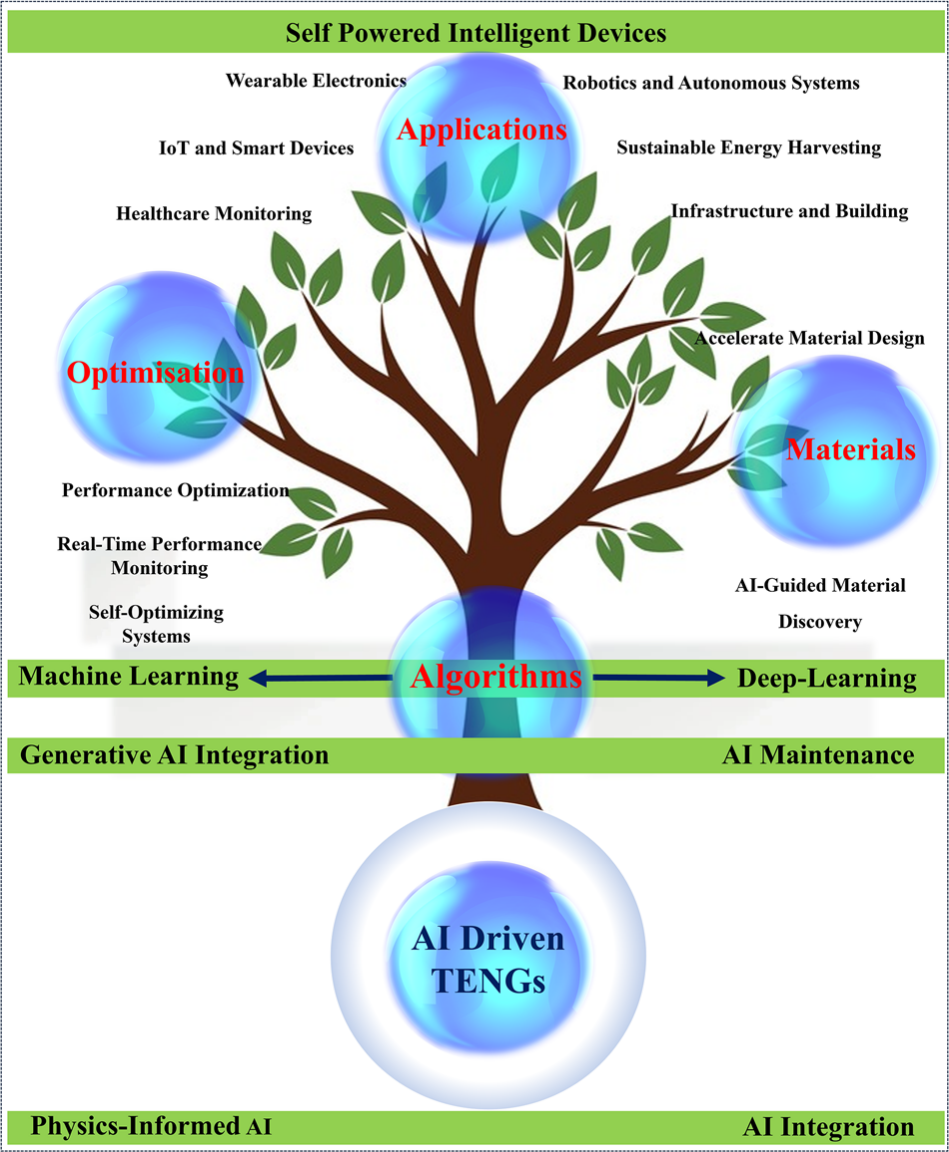
An in‐depth overview of this review article, highlighting AI's fundamental role in optimizing and advancing the functionality of TENGs.

**Figure** **2** provides an in‐depth overview of this review, highlighting AI's fundamental role in optimizing TENG functionality and advancing their practical applications across industries and particularly in healthcare,^[^ ^23^, ^24^ ^]^ environmental monitoring,^[^ ^25^ ^]^ industrial automation,^[^ ^26^ ^]^ and in Human‐Machine Interface (HMI).^[^ ^27^, ^28^ ^]^ The paper is organized into four main sections.

Section 2 introduces the algorithms utilized in AI for TENGs, providing a foundational understanding of their application. Section 3 delves into the development of AI‐enabled TENG systems, focusing on material selection, process optimization, and end‐use applications in industries, such as healthcare, wearable sports technology, smart homes, and human–machine automation. Finally, Section 4 presents an analytical discussion and offers recommendations, addressing challenges, and outlining the future scope of AI‐driven TENG innovations.

## 2 Algorithms Used in AI for TENGs

**Figure 3:**
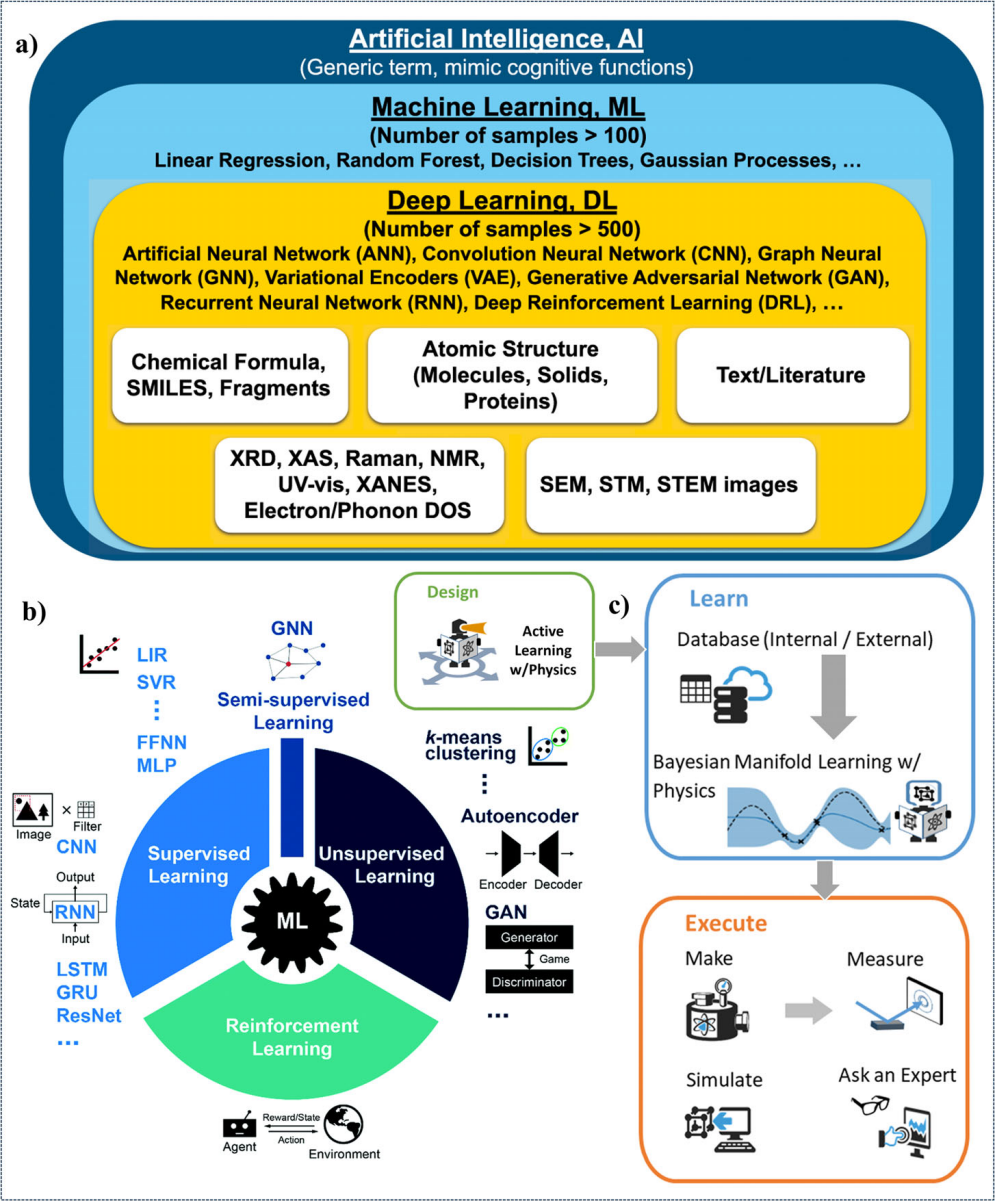
a) A comprehensive overview of the application of AI in materials science. (b) and (c) depict the AI workflow, from data collection and preprocessing to model training, evaluation, and deployment. Reproduced with permission.^[^ ^36^ ^]^ Copyright 2021, Royal Society of Chemistry,^[^ ^37^ ^]^ Copyright 2021, Springer Nature,^[^ ^38^ ^]^ Copyright 2022, Nature.

AI is developing intelligent systems to emulate human cognitive functions like learning, reasoning, and problem‐solving. AI is increasingly applied to developing TENGs for miniaturizing power sources used for energy harvesting, to enhance performance and optimize energy efficiency (see Figure 1c).^[^ ^7^, ^29^ ^]^ AI enables TENG systems to analyze real‐time environmental data, adapt power generation strategies, and predict maintenance needs, thereby boosting their efficiency and reliability across applications, including self‐powered sensors and wearable devices.^[^ ^30^, ^31^ ^]^ **Figure** **3** provides a detailed overview of AI's applications in materials science, emphasizing how AI, especially through ML and DL, supports data‐driven advancements. Figure 3a categorizes the AI landscape into different levels, showcasing ML and DL as powerful tools with unique algorithmic capabilities tailored for various materials science applications. ML techniques, such as linear regression, DT, and RF are suitable for medium‐sized datasets and pattern recognition tasks, while DL techniques, which require larger datasets, include NN architectures like Convolutional Neural Networks (CNNs) and Recurrent Neural Networks (RNNs), and are effective for complex, high‐dimensional data. Figure 3b,c illustrates a typical AI workflow for TENG research, which includes steps from data collection and preprocessing to model training, evaluation, and deployment, outlining a structured approach to materials discovery and design. This framework enables continuous TENG innovation by leveraging real‐world data to enhance performance. It begins with data collection, preprocessing, and feature extraction, allowing AI algorithms to analyze key metrics like voltage, current, power density, and environmental factors, such as temperature and humidity. This process supports real‐time adjustments and predictive insights for optimized performance.^[^ ^25^, ^29^, ^32^ ^]^

A key strength of AI in this context lies in pattern recognition and anomaly detection through supervised, unsupervised, and reinforcement learning techniques. Using labeled data, supervised learning enables TENG systems to predict energy output and adjust performance parameters to enhance reliability and stability. Unsupervised learning, by clustering unlabeled data, uncovers hidden patterns in TENG performance, supporting user behavior analysis and environmental monitoring. Reinforcement learning further enhances adaptability by enabling TENG systems to dynamically refine energy generation strategies in response to changing conditions.^[^ ^33^, ^34^, ^35^ ^]^

**Table** **1** provides a quick overview of AI algorithms applied in TENG systems, demonstrating their roles across various user applications, from texture recognition and gesture identification to driver action detection and gait analysis. Each application leverages different AI models, such as artificial neural networks (ANNs), convolutional neural networks (CNNs), and support vector machines (SVMs) achieving high accuracy rates in specific contexts, thereby illustrating the adaptability of AI to the unique requirements of TENG‐based applications.^[^ ^49^ ^]^ More advanced models, like graph neural networks (GNNs), RNNs, and optimization algorithms like genetic algorithms and particle swarm optimization, are highly valuable for analyzing complex, nonlinear data interactions, making them essential for refined energy optimization and sophisticated pattern recognition.^[^ ^50^, ^51^, ^52^ ^]^ Furthermore, optimization algorithms and fuzzy logic approaches enhance TENG efficiency by finding optimal configurations in complex solution spaces and allowing for approximate reasoning when data are noisy or incomplete.^[^ ^33^, ^34^, ^35^ ^]^ This adaptability is particularly beneficial in real‐world applications where environmental conditions are often variable and nondeterministic. The benefits of AI extend beyond TENGs to fields, such as drug discovery, healthcare analytics, and biomedical device design, where it automates complex analyses, optimizes processes, reduces operational costs, and supports data‐driven decision‐making.

**Table 1:** Overview of AI algorithms applied in TENG systems.

Sl. No.	Applications	Algorithms/models	Accuracy rates [%]	Refs.
1	Texture recognition	ANN	93.33	[39]
2	Handwriting recognition	SVMDT	99.66	[40]
3	Gesture identification	LSTM	82.3	[41]
4	Intelligent keyboard	DBN	98	[42]
5	Keystroke identification	SVM	98.7	[43]
6	User identification	ANN	99	[44]
7	Touchpad	CNN	93.6	[45]
8	Drivers action detection	RF	92	[46]
9	Character identification	DNN	89	[47]
10	Gait analysis	ANN	98.4	[48]

### 2.1 ML Algorithms for TENG

**Figure 4:**
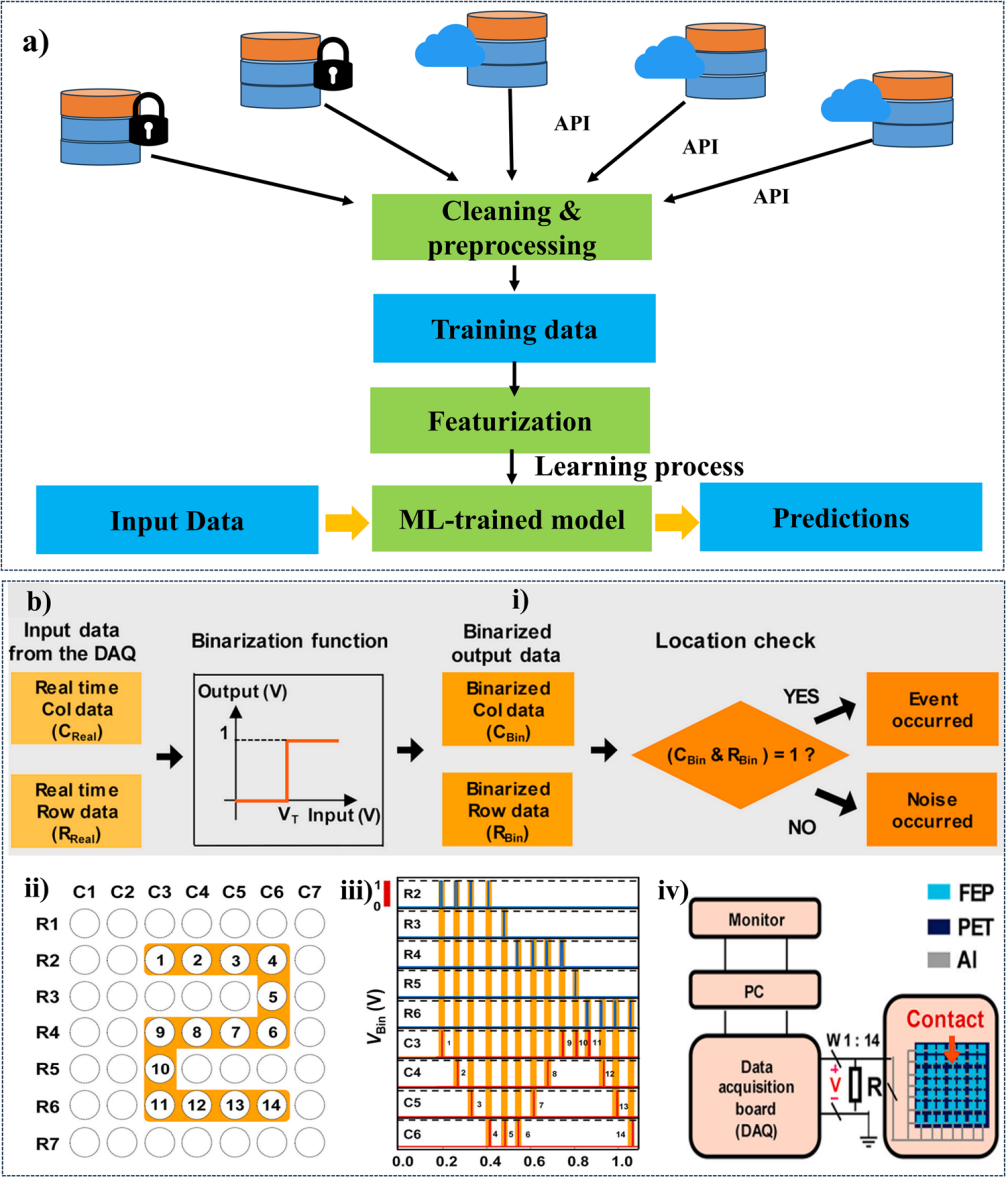
a) Illustrates the key steps in a typical ML workflow. Reproduced with permission.^[^ ^57^ ^]^ Copyright 2022, Frontiers. b, i) The procedure for data processing and the detection of touch points in each mode, along with the corresponding electrical signals. b, ii) Diverse hand‐written number patterns are represented through binarized data. b, iii) The multichannel measurement system is used to acquire information from the user's touch. Reproduced with permission.^[^ ^45^ ^]^ Copyright 2020, Elsevier.

ML is a subset of AI focused on developing algorithms that allow computers to learn from and make decisions based on data without being explicitly programmed. By identifying patterns within vast datasets, ML models enable systems to predict outcomes, make informed decisions, and continuously improve based on feedback. In TENG technology, ML is a transformative tool, offering sophisticated analysis capabilities that reveal intricate data patterns traditional methods often miss. Through training on extensive datasets, ML algorithms can predict TENG behavior across varying conditions, such as environmental changes and operational frequencies and optimize device parameters, like materials and electrode positioning, for maximum energy output. This adaptive ability has made ML invaluable for performance monitoring, anomaly detection, and diagnostics, facilitating proactive maintenance and improved reliability.^[^ ^53^, ^54^ ^]^ The programming languages for ML include Python, R, and MATLAB, with Python being the most widely used due to its extensive libraries like TensorFlow, PyTorch, and Scikit‐Learn. These libraries offer prebuilt functions for data manipulation, model training, and visualization, which streamline the development process. **Figure** **4**a provides an outline of a typical ML workflow tailored to TENG technology, covering data collection, preprocessing, feature extraction, and model training. After training, the model can predict new data patterns, offering insights for real‐time or near‐real‐time data analysis (Figure 4b). Specific steps include binarizing data, event detection, grid‐based data representation, and setting up hardware for data acquisition, ensuring that researchers can harness ML to identify events, anomalies, and patterns with precision. Through these methods, ML enables researchers to unlock the full potential of TENG technology, enhancing its design, operational efficiency, and manufacturing processes, ultimately driving the technology toward greater efficacy and innovation.^[^ ^55^ ^]^

In TENG systems, ML algorithms are crucial for optimizing energy harvesting and enhancing overall performance. For instance, SVMs play a key role in classification and anomaly detection, establishing decision boundaries critical for assessing TENG device performance and identifying deviations. This capability is essential for real‐time maintenance and energy generation optimization. A notable example is Li et al.,^[^ ^56^ ^]^ used SVM to achieve an 83.6% accuracy in distinguishing different operational states in a shaker system, showcasing SVM's precision and real‐time fault detection strengths. ANNs are another powerful tool that provides unmatched versatility across various TENG tasks. Their complex pattern recognition, predictive maintenance, and real‐time optimization capabilities significantly enhance system adaptability to dynamic conditions.

Zhang et al. demonstrated this by combining the fast fourier transform (FFT) with ANNs, achieving a remarkable 98.4% accuracy in patient identification based on triboelectric signals.^[^ ^48^ ^]^ For classification tasks, combining SVM with DT (SVMDT) balances interpretability and accuracy. Hang et al. used SVMDT for multiclass classification, achieving accuracies between 91.36% and 99.66% in handwriting recognition, which has applications in security and privacy.^[^ ^40^ ^]^ Additionally, algorithms like Mean Gaussian SVM, RF, and K‐NN further enhance TENG system performance. Mean Gaussian SVM handles nonlinear data relationships well, while RF, an ensemble learning method, boosts forecast accuracy, performance prediction, anomaly detection, and energy optimization. K‐NN's simplicity and effectiveness in classification make it invaluable for real‐time maintenance and issue identification.^[^ ^46^, ^58^ ^]^ Together, these ML algorithms empower TENG systems with greater intelligence, adaptability, and functionality. SVMs and ANNs enhance classification and anomaly detection, SVMDT provides a balance between interpretability and accuracy, and algorithms like RF and K‐NN contribute to real‐time decision‐making and predictive maintenance. Collectively, these tools enable TENG systems to excel across diverse applications, from wearables to IoT and renewable energy, pushing the boundaries of advanced energy harvesting capabilities.

#### 2.1.1 DL Algorithms for TENG

DL is a subset of ML that offers a powerful, data‐driven approach to predicting complex tasks and optimizing parameters in TENG systems, making it a transformative technology in this field.^[^ ^28^, ^59^ ^]^ The performance of a TENG device is influenced by various factors, including the materials used, the surface area of the electrodes, and the separation distance between them.^[^ ^28^, ^60^ ^]^ DL models can predict TENG output based on these parameters, which can then be optimized to achieve the desired performance.^[^ ^59^ ^]^ In the realm of TENG technology, multiple DL algorithms play critical roles in enhancing functionality. For example, a recent application integrates TENGs with a DNN to enable lip reading. This TENG device, designed with a layered structure of materials, such as nylon, PVC, Cu, and sponge, optimizes triboelectric charge generation. As it captures lip‐motion signals, the DNN processes these signals to accurately predict expressions, opening new possibilities for real‐time human–computer interaction. This innovative approach offers the potential to revolutionize interaction technology by enabling instant interpretation of lip movements. The model undergoes rigorous testing and validation with feedback loops that continuously improve its accuracy. Through these DL‐driven methods, researchers can optimize TENG parameters and unlock the full potential of TENGs for advanced energy harvesting and interactive applications.

**Figure 5:**
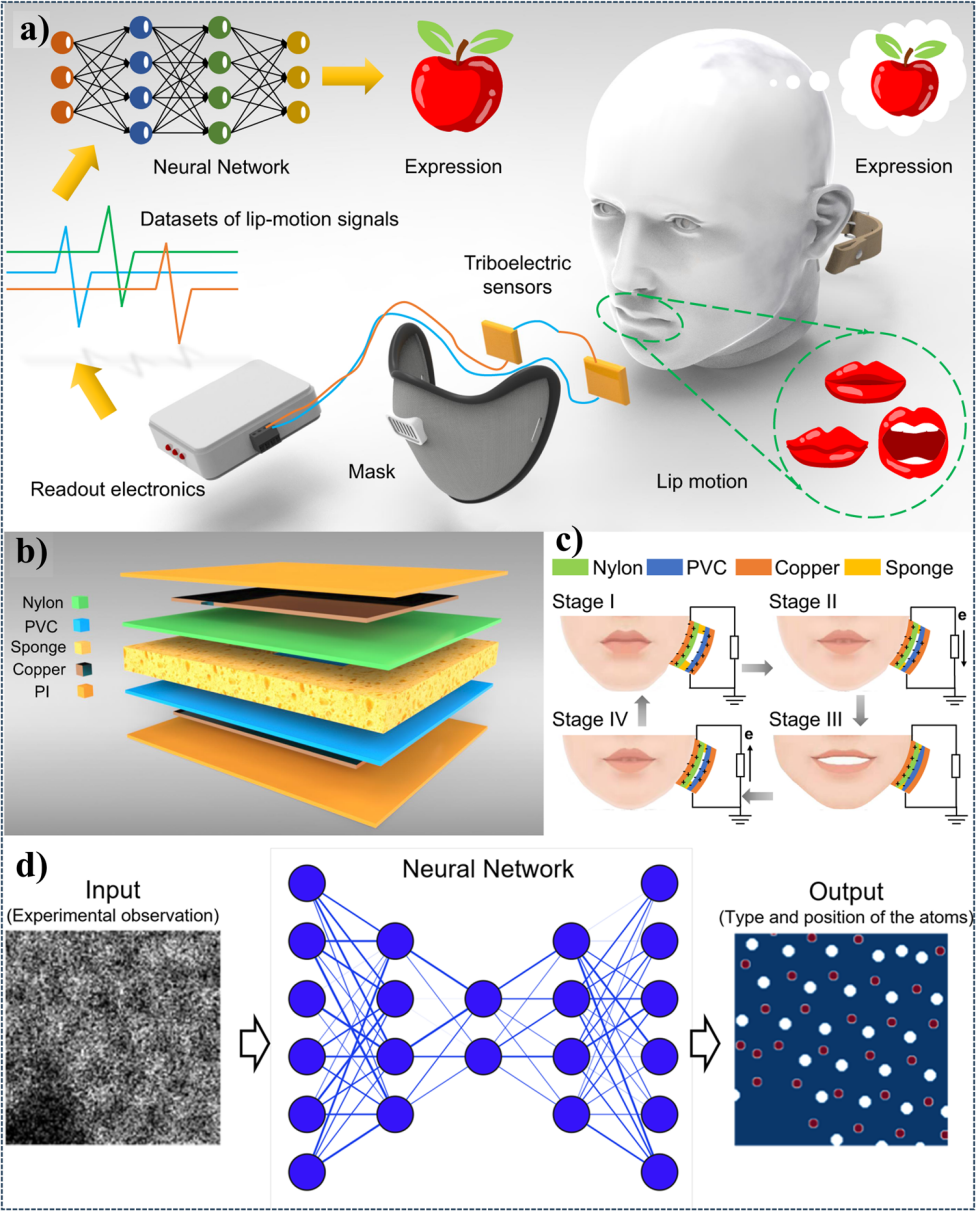
a) The system architecture, showcasing the acquisition of lip‐motion signals using TENGs, the training of a NN on datasets of these signals, and the subsequent prediction of expressions. b) Presents the layered structure of the TENG device, consisting of various materials, such as nylon, PVC, Cu, and sponge, to optimize triboelectric charge generation. c) Illustrates the different stages involved in the fabrication process of the TENG device, including material selection, layer assembly, and electrode placement. d) The input–output relationship of the DNN, where experimental observations of lip‐motion signals are used as input to train the network, and the predicted type and position of atoms are generated as output. Reproduced with permission.^[^ ^29^ ^]^ Copyright 2022, Elsevier,^[^ ^61^ ^]^ Copyright 2022, Nature,^[^ ^62^ ^]^ Copyright 2021, Nature.

In **Figure** **5**, the system architecture for this application is depicted, highlighting the process of acquiring lip‐motion signals through TENGs, training an NN on these data sets, and predicting expressions. The layered structure of the TENG device, made of materials like nylon, PVC, Cu, and sponge, is shown to optimize charge generation. The stages of device fabrication, from material selection and layer assembly to electrode placement, are also illustrated. Finally, the DNN's input–output relationship is depicted, showing how experimental lip‐motion data are used to train the network, which then predicts the types and positions of atoms as output.

In TENG applications, CNNs are essential for image and signal processing, significantly improving fault detection and quality control by identifying specific patterns and conditions in TENG‐generated signals. For instance, Wang et al.,^[^ ^63^ ^]^ developed a dual‐channel measurement method to enhance signal stability, enabling real‐time sensory signal analysis, particularly useful in applications like smart mat systems that require personnel identification, state monitoring, and precise positioning. Leveraging CNNs, TENG systems achieve higher accuracy and reliability, making them adaptable for complex, real‐world applications DNNs further add versatility to TENG applications by excelling in complex pattern recognition, predictive maintenance, and real‐time optimization. Jiang et al.,^[^ ^29^ ^]^ used a novel DNN‐based AI algorithm to predict TENG performance across diverse structures and configurations, providing a broader understanding of underlying data patterns by forecasting power output in unexplored parameter ranges. Stochastic gradient descent (SGD), while not a DL algorithm, is essential for efficiently training NNs by ensuring accurate model convergence within TENG systems. Jiang et al., applied advanced AI techniques with SGD optimization to refine parameters at the micro‐ and nanometers scale, offering insights into how structural parameters impact output power and enhancing predictive capabilities for sliding‐mode TENGs under various load conditions.^[^ ^29^ ^]^ Additionally, RNNs and long short‐term memory (LSTM) networks are crucial for handling sequential data and time‐series analysis, enabling predictions related to energy generation, wear and tear modeling, and optimization based on historical data in TENGs.^[^ ^64^ ^]^ These DL algorithms collectively improve TENG systems' intelligence and efficiency, ultimately enhancing energy harvesting, predictive maintenance, and overall performance across diverse applications.

As DL technology advances, new and sophisticated algorithms promise to further enhance TENG performance. Emerging DL approaches have the potential to unlock previously inaccessible efficiency, design, and functionality improvements for TENGs. DL algorithms bring distinct advantages to TENG analysis by capturing complex data relationships accurately, improving predictive accuracy, and providing valuable insights. These algorithms also manage large datasets effectively, a crucial feature considering the vast amount of data generated by TENG devices. The automation capabilities of DL streamline data analysis, reducing the time and effort involved in research and development. However, challenges remain, particularly in acquiring large, well‐labeled datasets for TENG analysis, which can be both costly and complex. Ensuring accurate data labeling also poses a significant hurdle. Despite these challenges, DL continues to drive meaningful advancements in TENG technology, paving the way for more intelligent, efficient, and responsive energy solutions.

## 3 AI‐Enabled TENG Systems

AI‐driven TENG systems are transforming energy harvesting by integrating AI to optimize each phase of the TENG lifecycle, from material selection to intelligent control and real‐time operation. ML and DL models can analyze extensive datasets during the design stage, allowing researchers to identify optimal materials and structures that maximize energy output. This data‐driven approach accelerates material selection, enhancing TENG's adaptability to specific applications, by tailoring designs to operational needs. During operation, AI empowers TENGs to dynamically adjust to environmental factors like temperature fluctuations or mechanical stress by modifying parameters, such as load resistance and electrode configuration. This real‐time adaptability ensures efficient performance across varying conditions. Beyond optimizing output, AI enhances energy management by predicting demand, conserving power when needed, and prioritizing critical tasks. This adaptability is particularly beneficial for applications like health monitoring devices or environmental sensors, where it extends battery life, improves reliability, and reduces maintenance. Predictive maintenance further extends the TENG lifespan by enabling AI algorithms to detect anomalies early, thereby preventing potential faults and minimizing downtime. In essence, AI allows TENGs to function intelligently and autonomously adapting to user behavior and environmental changes. This fusion holds vast potential for self‐sustaining, efficient energy solutions in sectors, such as healthcare, smart cities, and environmental monitoring, setting a new standard for sustainable energy innovation.^[^ ^65^, ^66^, ^67^ ^]^ This section critically analyses how AI is applied in TENG material selection, synthesis, optimization, and intelligent applications.

### 3.1 AI in Material Selection and Fabrication

**Figure 6:**
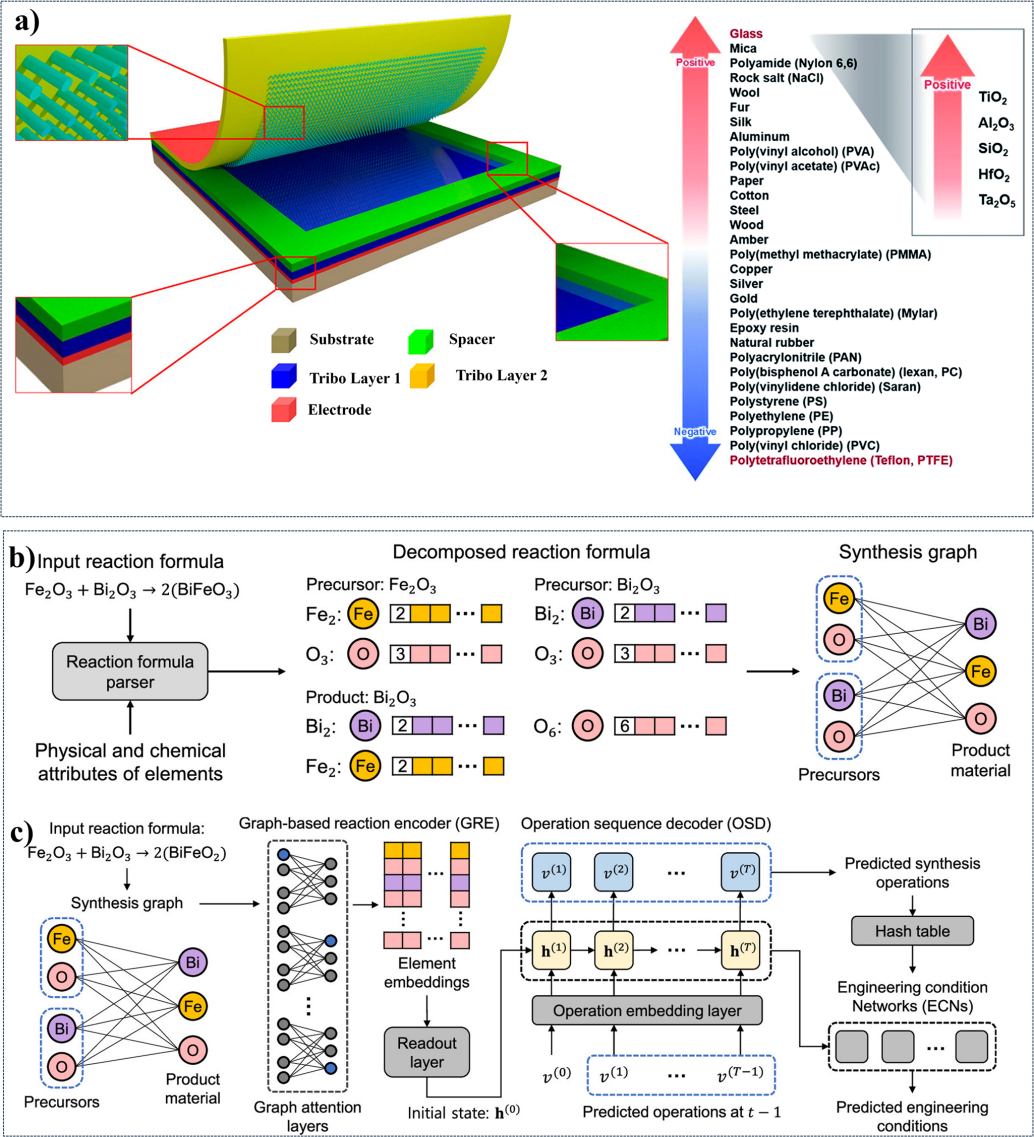
a) A schematic illustration of a TENG that highlights its essential elements. b) Shows a synthesis graph, the input reaction formula, and the decomposed reaction formula for the chemical reaction that occurs during material synthesis. c) The use of ML for chemical reaction prediction using an OSD and a GRE. Reproduced with permission.^[^ ^81^ ^]^ Copyright 2023, American Chemical Society,^[^ ^82^ ^]^ Copyright 2012, American Chemical Society,^[^ ^83^ ^]^ Copyright 2017, Royal Society of Chemistry.

The selection of materials for TENGs is crucial for optimal performance. The transfer of electrons between materials, known as triboelectrification, is influenced by properties, such as elasticity, friction, and surface texture.^[^ ^66^, ^67^ ^]^ Common electron acceptor materials include polytetrafluoroethylene (PTFE),^[^ ^68^ ^]^ polydimethylsiloxanes (PDMS),^[^ ^69^ ^]^ polyvinylidene fluoride (PVDF),^[^ ^70^ ^]^ fluorinated ethylene propylene (FEP),^[^ ^71^ ^]^ and Kapton,^[^ ^72^ ^]^ with PTFE and PDMS being the most widely used. PTFE offers high chemical and thermal stability, while PDMS is known for its elasticity and flexibility. Metal films, like Al,^[^ ^73^, ^74^ ^]^ Cu,^[^ ^75^ ^]^ Ag,^[^ ^76^ ^]^ and Ni^[^ ^77^ ^]^ are frequently used as electrodes and triboelectric layers. For example, Lie et al., developed a flexible TENG using an FEP sheet and a Ni mesh, achieving significant voltage and current outputs.^[^ ^77^ ^]^ Nanomaterials like carbon nanotubes (CNTs), metal‐organic frameworks (MOFs) and graphene can significantly improve the performance of TENGs. CNTs offer excellent electrical conductivity and high mechanical strength, while graphene provides high electrical and thermal conductivity. There's been a growing interest in exploring alternative materials, particularly functionalized materials, inorganic materials, and eco‐friendly options. Cellulose‐based materials, such as nano‐ and microcellulose, regenerated cellulose, and modified cellulose, have gained attention due to their sustainability. Kim et al., successfully used cellulose nanofibers as a dielectric layer in TENGs, resulting in a notable power output density.^[^ ^10^, ^78^ ^]^ In **Figure** **6**a, the layered structure of a TENG is depicted, showing its primary components: substrate, spacer, two triboelectric layers, and electrodes. The triboelectric layers are constructed from materials with different electron affinities, arranged in a way that facilitates electron exchange upon contact and separation, driven by mechanical force. This electron transfer creates an electric potential difference, thereby generating energy. The accompanying chart categorizes materials from positive to negative based on their electron affinity, indicating options for selecting triboelectric materials with complementary properties for maximum energy output. Researchers can improve power output, efficiency, and durability by adding nanomaterials (nanofibers, MOFs, etc.) to TENG structures.^[^ ^79^, ^80^ ^]^ Even though the choice of triboelectric materials has been extremely successful, many other materials are still untested.

To increase the use of TENGs, new materials, functionalized materials, inorganic materials, and composite materials are highly desired. For the development of the TENG model for upcoming self‐powered medical device applications, Gupta et al. suggested a solution‐processed ZnO‐Polystyrene (ZnO‐PS) composite. Because of the altered work function brought on by (PS) polymer diffusion during thermal annealing, the ZnO‐PS composite nanostructure produced a more enhanced surface charge. The output voltage of the ZnO‐PS TENG was higher (7 V) than that of the pure ZnO TENG (5.8 V).^[^ ^68^ ^]^ Similarly, Kim et al., developed a flexible TENG using PDMS‐CNT nanostructures. By incorporating CNT nanostructures, the PDMS‐CNT film increased the charge density on the polymer surface. The resulting TENG demonstrated a significantly higher output voltage (77.8 V) compared to pure PDMS and PDMS‐CNT composite.^[^ ^84^ ^]^ These developments demonstrate how nanomaterials can be used to improve TENG performance. Researchers can quickly find optimal material combinations for effective energy harvesting by using AI to analyze large datasets and simulate complicated material interactions. This procedure saves a large amount of time and resources compared to standard experimental approaches. By analyzing massive datasets, AI can predict material properties, identify optimal synthesis conditions, and build unique materials with desired properties.

Li et al. introduced a cost‐effective approach to enhance the performance of PDMS‐based TENGs by incorporating PTFE emulsion. This composite film, PDMS‐PTFE, significantly boosted the output voltage and charge transfer compared to pure PDMS. The optimal PTFE content of 10% weight resulted in a threefold increase in output voltage and current.^[^ ^85^ ^]^ A potential benefit of deploying Eu‐doped PVDF nanofibers as an active layer for TENGs was investigated by Kim et al. The electrical output power of the TENGs was significantly increased by adding Eu^3+^ ions. The TENGs' output voltage, current, and power density all arise in conjunction with the Eu content. However, performance deteriorated because of over‐doping.^[^ ^74^ ^]^ Here, in such conditions, AI can help to optimize the doping process by predicting the best concentration for maximum performance. ML algorithms can predict the impact of different doping levels on factors like charge generation, carrier mobility, and dielectric properties, enabling researchers to fine‐tune the material composition for optimal performance. Training on this data allows the AI model to determine the ideal doping level that maximizes performance by avoiding the drop due to excessive doping. This method can significantly speed up the optimization process while reducing the number of experimental trials necessary.

AI has the potential to significantly accelerate fabrication processes along with material selections, streamline inspections, and enable continuous improvement throughout a product's lifecycle. This technology offers a wide range of applications, from optimizing specific manufacturing steps to integrating seamlessly into enterprise‐wide Industrial programs.^[^ ^86^ ^]^ ML is becoming more and more popular in materials science as a tool for analyzing large datasets and forecasting material characteristics. These databases frequently include details on the microstructure, intended behaviors, and composition of the materials.^[^ ^36^ ^]^ The objective is to develop ML models that can predict desired mechanical characteristics or behaviors in a timely and accurate manner. These models can even be able to find new material compositions or structures that perform better than those of currently available materials.^[^ ^87^ ^]^ Large datasets from experiments or simulations that investigate the connection between composition and characteristics are usually accessible for materials with complex and disordered microstructures, such as glasses and alloys.^[^ ^88^ ^]^ Relevant characteristics in these situations, including the proportions of various elements, might be expressed as feature vectors. Predicting material characteristics is especially well‐suited for ML techniques that are highly proficient at handling vector data.^[^ ^89^ ^]^ Gyoung's work makes a significant contribution to the field of materials science by proposing innovative AI‐driven methods for accelerating material discovery and development. The paper proposes a novel multimodal graph‐to‐sequence model to bridge the gap between material discovery and synthesis, which predicts the necessary synthesis operations and conditions based on a novel multimodal graph‐to‐sequence model. This model predicts the necessary synthesis processes and circumstances based on the chemical composition of the precursor and target materials. The algorithm was trained on a dataset of 771 thermoelectric materials and showed great accuracy in predicting synthesis processes. It can also build human‐readable synthesis recipes from huge language models. This technique provides a possible answer to technical issues in material synthesis, accelerating the development of high‐performance thermoelectric materials.^[^ ^81^ ^]^ The chemical processes that take place during a material's production are depicted in Figure 6b. It displays a synthesis graph, the input reaction formula, and the decomposed reaction formula.

The interactions between several precursors and the product material are depicted in the synthesis graph. ML method for forecasting chemical reactions is shown in Figure 6c. It uses an operation sequence decoder (OSD) and a graph‐based reaction encoder (GRE). The OSD forecasts the steps required to create the intended product, while the GRE converts the supplied reaction formula into a graph representation. Another work used support vector regression, least absolute shrinkage and selection operator (LASSO), RF, and multilayer perceptrons (MLP) algorithms to estimate Young's modulus in silicate glasses, exploring deeper into ML's applicability in materials science.^[^ ^90^ ^]^ The results indicated that MLP achieved the highest accuracy, while LASSO offered a slightly lower accuracy but provided a simpler and more interpretable model. The study also demonstrated that Gaussian process regression could be a viable alternative to NNs for datasets with limited data points to avoid overfitting.^[^ ^91^ ^]^

Wang et al. developed a NN‐based design system for Cu alloys. This system can rapidly search through various compositions and identify alloys with desired properties like tensile strength and electrical conductivity.^[^ ^96^ ^]^ In another study, Zhao et al., investigated how different ML models, dimensionality reduction techniques, and additional material features can be combined to effectively select strong and conductive Cu alloys. For materials with gradient nanostructures, researchers have developed Gaussian process‐based surrogate models. These models can predict how the structure's gradient affects its strength and deformation mechanisms.^[^ ^97^ ^]^ Wen et al. used ML models to predict high‐performance materials. They then synthesized these predicted alloys and achieved hardness values exceeding any material in the training data. This demonstrates the ability of ML to discover entirely new materials.^[^ ^98^ ^]^ ML can also be used to understand how a material's structure influences its properties. For instance, DNNs have been trained to predict the mechanical response of cellular materials based on their geometric patterns^[^ ^99^ ^]^

ML's applicability extends to various materials. Liu et al, employed ML to predict the fracture toughness of polycrystalline silicon.^[^ ^100^ ^]^ In another study, researchers used a NN to predict the strength and toughness of spider webs based on fiber properties and web connectivity.^[^ ^101^ ^]^ Hardian et al., highlights the potential of ML, to design sustainable materials. Traditional methods for material creation are often wasteful. The authors propose a new approach: using ML to design materials in computer simulations in silicon. Their method combines the established design of experiments with a custom‐built ML module. This module, containing elements like an SVM and an evolutionary algorithm, helps find the best synthesis parameters for materials. Importantly, it prioritizes minimizing environmental impact. The success of this approach is shown with the development of a sustainable metal‐organic framework (ZIF‐8) using electrochemical synthesis. The ML system efficiently analyses the complex relationship between synthesis conditions, material quality, and environmental impact.^[^ ^94^ ^]^ The authors stress that incorporating ML into material design greatly lowers waste and its negative effects on the environment in addition to increasing efficiency. They demonstrate how AI‐driven methods have the potential to transform the synthesis of sustainable materials by concurrently maximizing the number of objectives. Their research shows that integrating ML with simulations based on physics can result in the creation of more inventive and sustainable materials. Furthermore, Erfan et al., utilized AI to improve parts created with laser powder‐bed fusion (LPBF), a popular 3D printing technique. LPBF lets you build complex shapes, but the settings used during printing can affect the final part's strength and other properties. Here the researchers used a type of AI called a NN to analyze data on how different printing settings (laser power, speed, etc.) influence the strength and stretchiness of titanium alloy parts. By feeding the network existing experimental data, they pretrained it for accuracy. The NN then acted as a powerful tool to predict the best printing settings for creating parts with the desired strength. This approach has the potential to significantly improve the quality and performance of parts made with LPBF 3D printers.^[^ ^102^ ^]^ Along with this, AI can be employed with 3D printing processes, such as LPBF to form closed‐loop systems for continual optimization. Also, developing material‐specific AI models can improve accuracy and efficiency in material design and synthesis processes. Both Hardian et al.,^[^ ^94^ ^]^ and Erfan et al.,^[^ ^102^ ^]^ leverage the power of AI to address inefficiencies in different stages of material creation. While Hardian et al., focus on using ML to design sustainable materials in the early development stages, Erfan et al. apply AI to optimize the production processes for parts created through 3D printing. One of the most transformative contributions of AI and ML in material science is the substantial increase in the efficiency of material design.

**Figure 7:**
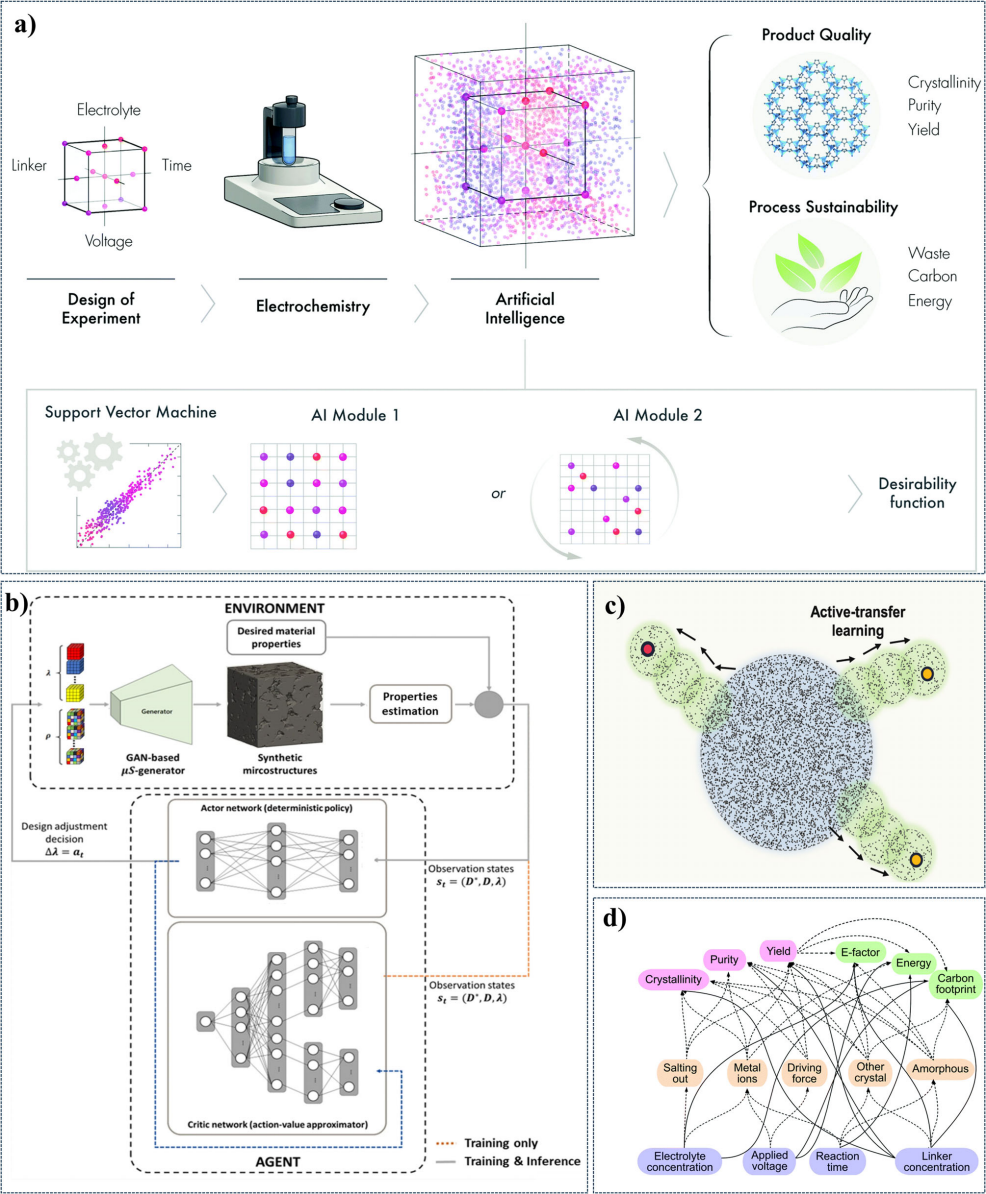
a) Standard procedures and process variables to assess ZIF‐8 electro‐organic synthesis. b) A schematic overview of morphology control via an AC model. c) Schematic of gradual expansion of reliable prediction domain of DNN based on the addition of data generated from the hyper‐heuristic genetic algorithm and active transfer learning. d) DNN's prediction domain is expanded through the addition of data generated by a hyper‐heuristic genetic algorithm and active transfer learning. Reproduced with permission.^[^ ^92^ ^]^ Copyright 2021, Nature,^[^ ^93^, ^95^ ^]^ Copyright 2022, Nature,^[^ ^94^ ^]^ Copyright 2020, Royal Society of Chemistry.^[^ ^95^ ^]^

Traditionally, exploring new materials required labor‐intensive experimentation and simulations, which are often impractical for navigating the vast design space. A novel approach to autonomous material synthesis, using AI‐guided decision‐making algorithms, was developed by Robert et al. demonstrating the power of AI‐driven optimization. Their team built a surrogate model from over 1000 experiments to simulate a robotic material synthesizer, with experimental campaigns validating the model's accuracy. The model was used to evaluate more than 150 AI‐guided strategies within a reinforcement learning framework, ultimately identifying an ensemble NN‐based strategy that outperformed traditional methods. This approach proved effective in navigating complex material synthesis environments, significantly accelerating material development while optimizing multiple parameters simultaneously. This advancement underscores the potential of AI to revolutionize material science by enabling faster, smarter, and more efficient pathways to material discovery and optimization.^[^ ^103^ ^]^ Conventional material discovery techniques depend on laborious physical testing and trial‐and‐error trials that take a lot of time and money. AI, on the other hand, speeds up this process by examining big datasets, finding trends, and modeling molecular‐level material interactions. As a result, less laborious laboratory tests are required, freeing up researchers to concentrate on the most promising material combinations. Furthermore, by selecting the top candidates before physical validation, AI‐driven predictive modeling reduces material waste and experimental expenses. High‐throughput simulations also make it possible to screen novel materials quickly, which drastically reduces the amount of time needed for research. Researchers can improve TENG optimization more quickly and affordably by using AI, which will eventually increase the efficiency of energy harvesting. **Figure** **7**a provides standard procedures and process variables to assess ZIF‐8 electro‐organic synthesis product quality and process sustainability and a simplified flow diagram showing how ML and applied AI modules are used. Figure 7b an actor‐critic model is employed to control the morphology of synthetic microstructures, iteratively adjusting parameters to achieve desired outcomes. The model learns through interaction with an environment that provides feedback on physical properties. Figure 7c the model's prediction domain is expanded by incorporating data from a hyper‐heuristic genetic algorithm and active transfer learning. And Figure 7d DNN's prediction domain is expanded through the addition of data generated by a hyper‐heuristic genetic algorithm and active transfer learning.

### 3.2 AI in TENGs Device Optimization

Optimizing the layered structure and design of TENGs is vital for maximizing energy output and ensuring stability in real‐world applications. Each component—electrodes, triboelectric layers, spacers, and substrates—significantly influences the efficiency of energy conversion. Enhancements in structural elements, such as layer thickness, surface patterning, and flexibility, improve durability and mechanical resilience, allowing for consistent performance over extended periods. Integrating AI further elevates TENG efficiency and adaptability by dynamically adjusting operating parameters to maximize energy harvesting from diverse sources, like human motion and environmental factors. Through ML, AI fine‐tunes energy conversion parameters, enabling TENGs to respond intelligently to changing conditions and load requirements. AI also plays a critical role in predictive maintenance, pattern recognition, and problem identification, which enhances performance and minimizes downtime.^[^ ^19^, ^104^ ^]^ AI algorithms play a transformative role in enhancing TENG performance by enabling sophisticated data analysis, improving energy conversion efficiency, and providing deeper insights into device behavior.^[^ ^1^, ^105^, ^106^ ^]^

AI's advanced capabilities in pattern recognition and anomaly detection are invaluable, as they can identify subtle trends and detect irregularities in TENG structures using ML techniques, such as supervised and unsupervised learning. This ability to pinpoint anomalies—like sudden drops in energy output—enables timely maintenance and troubleshooting, preventing potential issues before they escalate and ensuring reliable, long‐term operation of TENG systems.^[^ ^19^, ^49^, ^107^ ^]^ Khorsand et al., introduced a combined mathematical modeling and optimization approach to predict how structural parameters affect TENG performance, using a co‐evolutionary particles swarm optimization (CPSO) method to identify optimal design variables efficiently. Although effective, this approach is tailored to specific structural configurations and lacks broader applicability. To optimize key TENG parameters, such as contact area, film thickness, and external resistance, they applied the Gray Wolf algorithm, analyzing the relationship between these parameters and energy output to design high‐performance, lightweight TENGs. Experimental validation supported the simulation results, showcasing the effectiveness of this AI‐driven optimization strategy. However, the development of a more universal predictive model remains essential for broader TENG design and optimization, allowing for more versatile and adaptable TENG systems across various applications.^[^ ^108^ ^]^

Recent advancements in TENG research have prioritized boosting output performance, enhanced conversion efficiency, and optimizing structural designs. AI algorithms have become powerful tools for addressing these challenges, enabling breakthroughs in predicting energy output, optimizing design parameters, and even enabling applications like handwriting recognition through TENG‐powered devices. Jiang et al. developed the DNN‐based AI model to predict TENG performance accurately. This model demonstrated remarkable accuracy in forecasting the power output of various TENG structures, including grating, disc, and rolling configurations, closely matching experimental results. The DNN model, refined with the SGD technique, was further used to predict TENG performance across unexplored parameter ranges, providing valuable insights into the effects of structural characteristics on energy generation. This AI‐driven approach offers a more cost‐effective and efficient pathway for TENG design, surpassing traditional methods.^[^ ^29^ ^]^ Similarly, Ravi et al. investigated the use of ML techniques, notably K‐NN and NN to analyze actual energy data from TENGs. Their study predicts system accuracy and evaluates output quality in contact‐separation mode with varying load factors. The investigation includes training and testing ML models using real‐world data and evaluating their performance using measures, such as accuracy, recall, and F1‐score.^[^ ^58^ ^]^ The authors suggest that further advancements in AI for TENG research could involve exploring sophisticated DL techniques, such as CNNs or RNNs to enable more complex feature extraction and time‐series analysis of TENG data. Although AI‐driven TENG research is greatly advanced by these methods, it appears that hybrid models that incorporate the advantages of several algorithms might lead to even greater advancements. Incorporating real‐time adaptive learning processes can further enhance the accuracy and dependability of the model in dynamic settings.

**Figure 8:**
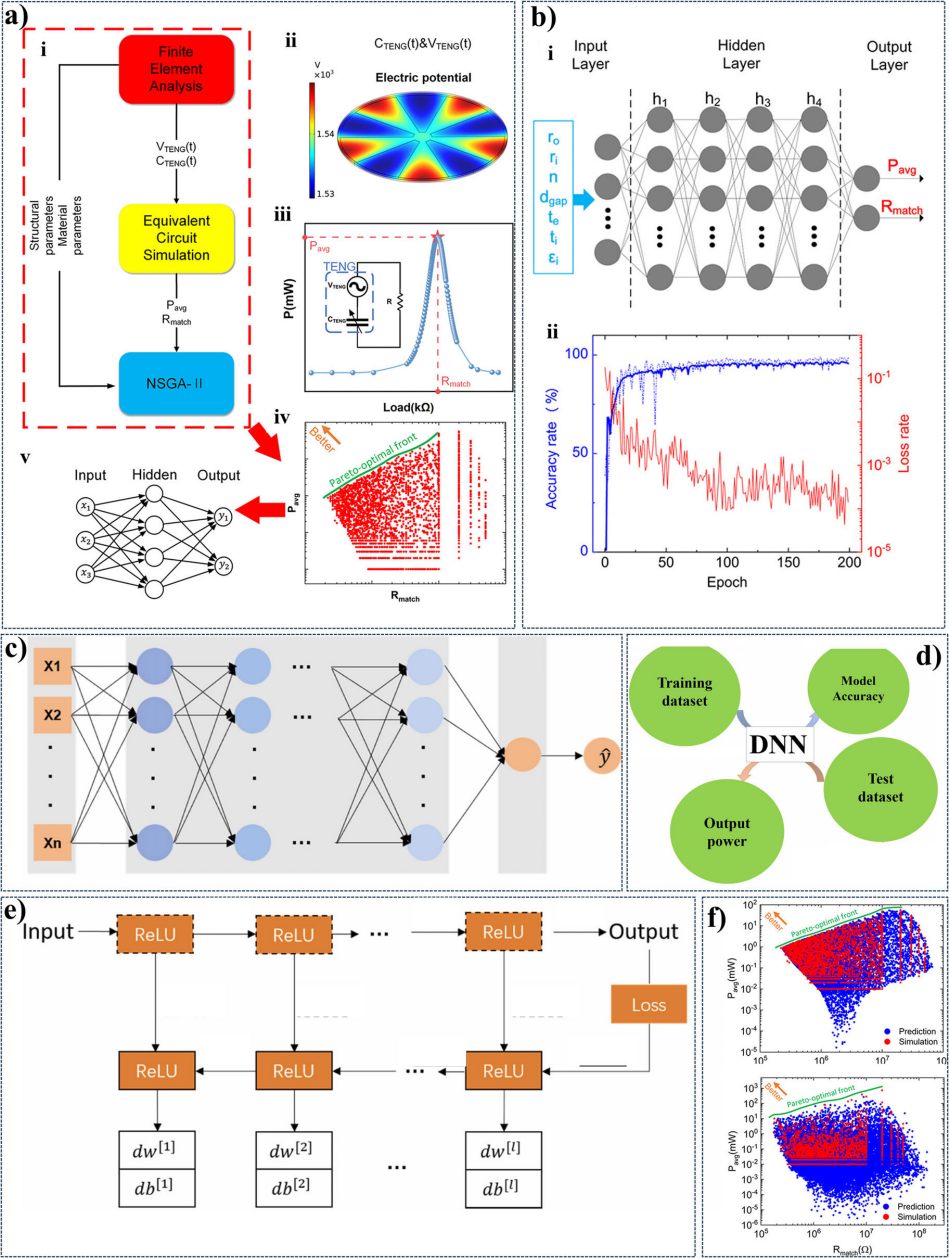
a) Depicts the overall framework, highlighting the integration of AI techniques for optimization, equivalent circuit simulation, and performance optimization. b) Demonstrates the use of an NN to predict the electric potential of a TENG based on input parameters. c) Illustrates the architecture of a DNN used for modeling and prediction. d) Presents the training and validation process of the DNN, showcasing the accuracy and loss rates. e) Details the backpropagation algorithm used to update the weights and biases of the DNN and f) Visualizes the relationship between the predicted and actual values of the output parameter, demonstrating the model's performance. Reproduced with permission.^[^ ^19^, ^29^, ^109^ ^]^ Copyright 2021, 2022, Elsevier,^[^ ^19^, ^29^ ^]^ Copyright 2024, Wiley.^[^ ^109^ ^]^^[109]^

**Figure** **8** illustrates the application of AI in optimizing TENG devices. The overall framework, depicted in Figure 8a, demonstrates the integration of AI techniques for equivalent circuit simulation, performance optimization, and prediction. Figure 8b showcases the use of a NN to predict TENG electric potential. The DNN architecture, visualized in Figure 8c, is trained and validated using backpropagation Figure 8d to learn from data and improve predictions. Figure 8f demonstrates the model's performance by comparing predicted and actual output values. AI holds significant potential in advancing TENG layer fabrication and device optimization, particularly in areas where research is still limited. Despite the promising capabilities of AI, there has been little work reported on its application in optimizing TENG devices, making this a fertile ground for further research and innovation. Developing AI‐based systems for TENG layer fabrication could help maintain consistent standards in production, ensuring quality and uniformity across devices. Data dependability can indeed be impacted by variations in ambient factors, device performance, and material qualities. It might be difficult to get reliable, high‐quality data to make sure AI helps with TENG optimization. Building a trustworthy dataset can be challenging due to the wide variations in material qualities, device performance, and ambient conditions. Variations in experimental setups and measuring settings might result in disparities, making inconsistent data gathering methods a major difficulty. This can possibly be addressed by employing automated measuring equipment and standardizing data gathering procedures, which can assist in enhancing uniformity and lower mistakes. Since TENG research is still in its infancy, another problem is the scarcity of huge datasets. This gap can be closed with the use of cooperative initiatives, data‐sharing websites, and AI‐powered data augmentation methods. Altogether, integrating AI in these aspects would not only improve the reliability of TENG performance but also accelerate research and innovation by providing a more rigorous, standardized framework for evaluating device outcomes.

### 3.3 AI in Intelligent Sensing Applications

AI uses training data to identify relationships between inputs and outputs through algorithms and statistical models, allowing computers to perform tasks without explicit instructions. It has been applied across fields like materials science, structural optimization, data analysis, and signal recognition.^[^ ^110^ ^]^ Its ability to handle noisy or incomplete data with flexibility and symbolic reasoning makes AI a valuable tool for solving practical challenges. AI models offer fast, accurate generalization, and prediction, making them an integral part of modern integrated systems and a powerful alternative to conventional methods.^[^ ^111^ ^]^ This ability of AI to analyze complex, nonlinear data, and make accurate predictions complements TENG's innovative approach to energy harvesting from mechanical movements. This synergy allows for real‐time adjustments and improved performance in various applications, from wearable electronics to self‐powered energy solutions.^[^ ^112^ ^]^ As TENGs are becoming a sustainable and reliable power source for IoT devices, enabling them to operate in remote or challenging environments is important.^[^ ^113^ ^]^

**Figure 9:**
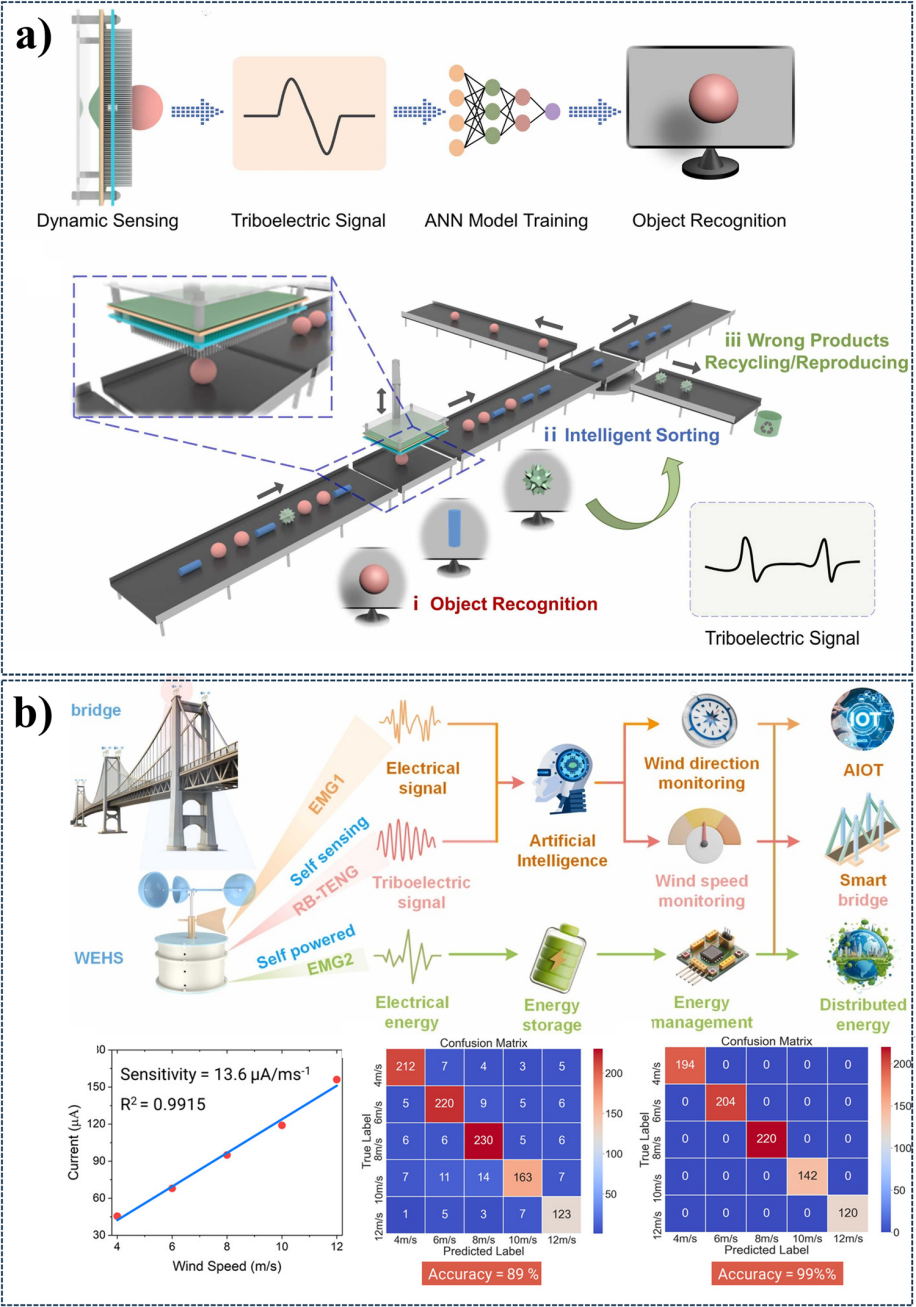
a) Illustrates a self‐powered intelligent sorting system. The system employs a NN model to recognize objects based on their triboelectric signatures. This enables the system to sort objects into different categories intelligently, reducing manual labor and improving efficiency. b) The image showcases a self‐powered wind energy harvesting and monitoring system. The system also employs an AI‐powered monitoring system to analyze the collected data and provide real‐time insights. This innovative approach demonstrates the potential of TENGs in sustainable energy harvesting and smart infrastructure. Reproduced with permission. Copyright 2022, Wiley,^[^ ^25^ ^]^ Copyright 2024, Elsevier.^[^ ^114^ ^]^

Intelligent devices that combine TENG technology with AI have the potential to revolutionize various aspects of our daily lives. These devices leverage the energy‐harvesting capabilities of TENG and the cognitive and decision‐making capabilities of AI to enhance functionality and efficiency in multiple domains. This combination allows for continuous data collection, remote monitoring, enhanced efficiency, and the development of innovative applications.^[^ ^18^ ^]^ AI‐based energy harvesting with TENG is a transformative approach that harnesses the power of AI to optimize energy generation. AI algorithms continuously monitor TENG device performance and environmental conditions, dynamically adjusting parameters to maximize energy output in real‐time. This results in efficient and adaptive energy harvesting, reducing downtime, extending device lifespan, and enabling predictive maintenance. AI also detects anomalies and offers data‐driven insights, guiding decision‐making for TENG system improvements. Moreover, the integration of AI with TENG systems enhances user experiences, personalizes energy generation, and supports sustainable and intelligent energy solutions across various applications. The convergence of AI and TENG is paving the way for innovative solutions in smart sensors, IoTs, HMIs, industrial automation, and smart transportation and infrastructures. From smart sensors that monitor industrial equipment and environmental conditions to IoT networks that optimize resource usage, AI‐based TENGs provide innovative solutions that enhance efficiency and sustainability **Figure** **9**.

TENG technology combined with AI in healthcare provides a constant power source for implantable medical devices, eliminating the need for battery replacement and improving patient care.^[^ ^115^ ^]^ Furthermore, in HMI, TENGs enable responsive, touchless control systems that improve user experience and safety. In industrial automation, AI‐driven TENG sensors facilitate predictive maintenance and quality control, contributing to more reliable and cost‐effective manufacturing processes.^[^ ^116^ ^]^ Moreover, in the context of smart cities, AI‐based TENG technology supports intelligent infrastructure and transportation systems that improve urban living conditions and infrastructure resilience. The adoption of AI‐based TENG systems across industrial and urban environments holds the potential for a future characterized by excellent connectivity, energy efficiency, and environmental sustainability becomes increasingly attainable. ML, as a fundamental component of AI, provides an effective approach for analyzing complex, multidimensional datasets generated by triboelectric‐based intelligent sensing systems. By integrating ML algorithms, such as SVM, DL, CNN, and RNN, these TENG‐based self‐powered sensing systems can not only optimize data analysis but also improve real‐time decision‐making and adaptability. This synergy enhances energy‐efficient sensing applications, such as health monitoring, human–machine interfaces, and gesture recognition, making these technologies more responsive and intelligent. The adoption of AI‐based TENG systems across industrial and urban environments holds the potential for a future characterized by excellent connectivity, energy efficiency, and environmental sustainability becoming increasingly attainable.^[^ ^13^ ^]^ **Tables** 2, 3, 4, 5, 6, 7 summarize advancements in AI‐enhanced triboelectric sensing systems developed for diverse applications including sensing and IoTs, HMIs, healthcare, sports/athletics, industrial automation, and smart city solutions. The applications of this technology highlight its transformative impact on modern society, underscoring the importance of sustainable innovation in addressing current challenges and building a sustainable foundation for the future.^[^ ^112^ ^]^

**Table 2:** AI‐enhanced TENG for intelligent sensor and IoT applications.

Sl. No.	Triboelectric materials	Mode ofoperation	AI Techniques/algorithms	Accuracy	Applications	Refs.
1	Polyester & Kapton	Contact separation	CNN	99.07% and 99.32%	Triboelectric tactile sensor for simultaneous material and texture recognition	[128]
2	PVDF	Contact separation	CNN	96.82%	Hybrid triboelectric‐inductive tactile sensor for object recognition	[129]
3	Aramid fibers & silicone	Contact separation	SVM	90.38%	Triboelectric sensor for object recognition	[130]
4	Microcone hydrogel & nano wrinkled PET	Contact separation and sliding	CNN	95% and 97.2%	Multifunctional hydrogel‐based E‐skin for material and texture recognition	[131]
5	PTFE and steel pillar	Contact separation	ANN and SVM	96.39 % and 87.5 %	Real‐time object recognition system	[115]
6	PVDF spun film and PVDF piezoelectric film	Contact separation	CNN	94.16%	Piezo‐tribo hybrid sensor for gestures recognition and controls	[132]
7	Natural latex & PTFE	Contact separation	AAL	95.41%	Triboelectric wristband for gestures recognition	[123]
8	PDMS‐coated Ni@C tube	Contact separation	CNN	99.8%	Textile tactile sensor for material and gesture recognition	[133]
9	Janus structured SSCNT/BC/WPU film & EcoFlex	Contact separation	MLP	98.63%	Tactile sensing system for material identification	[134]
10	PTFE & Cu	Contact separation and sliding	GRU	100%	Self‐sensing and self‐powered wearable system for human motion energy harvesting	[135]
11	CNT/Egg shell membrane, Cu and PEDOT: PSS	Contact separation	MLP	98.9%	Pressure‐sensitive E‐skin for real‐time material perception	[136]
12	Seaweed & Agar hydrogel	Contact separation	DL	100%	Self‐powered body area sensor network system for infant movement tracking	[65]
13	PET & FEP	Tapping	DL	93.6%	Transparent touchpad array for digit pattern recognition	[45]
14	PDMS & Cu	Contact separation	ML	99.66%	Multilanguage‐handwriting recognition	[40]
15	Hydrogel	Contact separation	1D‐CNN	98%	Strain sensor for hand‐written digit recognition	[137]
16	PDMS	Compression	Medium Gaussian SVM	93.5%	Self‐powered intelligent writing pad for letter identification	[31]
17	PA 66	Contact separation	CNN, LSTM, and GRU	99%	3D printer triboelectric sensor gloves for sign language prediction	[138]
18	AF/VMT paper & PDMS	Contact separation	ML	99.8%	Triboelectric sensor for human motion prediction	[139]
19	CNTs/TPE & EcoFlex	Contact separation	ML	96.7%	Human motion monitoring and gesture recognition gloves	[122]

**Table 3:** Application of AI‐enhanced TENG in urban safety and monitoring in smart city solutions.

Sl. No.	Triboelectric materials	Mode of operation	AI techniques/ algorithms	Accuracy	Applications	Refs.
1	PET & PVC	Contact separation	DL‐assisted data analytics	85.67%	Floor monitoring system for smart home applications	[64]
2	Al & Kapton	Contact separation	RF	92%	Driver's steering action detection for intelligent driving	[46]
3	PDMS yarn	Contact separation	Data analysis	≈100%	Identity recognition carpet for safeguarding entrance and early warning of intrusion	[194]
				≈	Intelligent footwear system for human motion monitoring and remote emergency rescue	
4	FEP & PVC	Contact separation	DL	94.44%	Smart mat system for personnel monitoring toward maritime safety	[63]
5	PVDF core‐sheath yarn‐based fabric	Contact separation	Classification coding and RNN	96.5%	Smart switch, smart infrared remote controller and material perception	[121]
6	PTFE & Cu	Contact separation and sliding	CNN	93.5%	Self‐powered smart floor for position sensing and personal identification	[195]
7	FEP & Wood	Contact separation	Data processing	–	Wireless gas sensor system for real‐time assessment of food quality	[196]
8	PET & PTFE	Contact separation	WGAN	81.06%	Self‐powered flexible sensors for smart transportation monitoring	[190]
9	PTFE and Cu	Contact separation and sliding	RNN	98.21%	Electromagnetic‐triboelectric wristband sensor for driver behavior detection system	[197]
10	Silicone rubber & water	Contact separation	LSTM	97%	Smart belt for high‐security double lock system	[198]
11	Tire tread, PI film, and LIG electrode	Contact separation and freestanding	STFT and CNN‐LSTM	95%	Tire monitoring system for self‐powered driving information recognition	[199]
12	Ag coated Nylon & Chenille yarn	Contact separation	CNN	83.3%	Triboelectric carpet fabric for motion monitoring and user identification	[200]
13	PLA & PTFE	Contact separation and freestanding	1D‐CNN	97.5%	Driver training assistance system for driver training and behavior monitoring	[201]

**Table 4:** Application of AI‐enhanced TENG interactive control within HMI systems.

Sl. No.	Triboelectric materials	Mode of operation	AI Techniques/ algorithms	Accuracy	Applications	Refs.
1	Graphene/SSG composites	Contact separation	Data analytics		Flexible smart touchless sensor	[145]
2	FEP	Sliding	Data analytics	–	Intelligent sliding unlock system as a biometric authentication system	[147]
3	PTFE, Ni fabric, TPU & EcoFlex	Contact separation and sliding	CNN	100%	Soft robotic perception system for object positioning and sensory cognition	[148]
4	Nylon & PTFE	Contact separation	ML	–	Gait monitoring system and gait password human identification system	[124]
5	PAN/BT & Ag	Contact separation	RF, ET, DT, and SVM algorithms	100%	International Morse Code on Motion Recognition	[144]
6	Graphene	Contact separation	Data analysis	–	Triboelectric touch‐free screen sensor for gesture‐recognizing	[140]
7	ZIF‐67 & Teflon	Contact separation	WT and WPT	–	Self‐powered robot object recognition and gait recognition	[149]
8	Silicone rubber & hydrogel film	Contact separation	LSTM	90%	Stretchable self‐powered sensor array for human–robot interaction and human motion monitoring	[150]
9	Acrylic elastomer PE	Contact separation	LSTM	97.2%	Humanoid Ionotropic skin for object recognition and sorting	[151]
10	EcoFlex and wrinkled nitrile	Contact separation	CNN	91.3%	Smart glove for sign language recognition and VR space bidirectional communication	[152]
11	PI & Ionic gel	Contact separation	MLP, CNN	92.3%	Micro pyramid array bimodal E‐skin for materials and surfaces shape perception	[153]
12	PDMS & Cu	Contact separation	NN	92.11%	Sensory receptor for real‐time neuromorphic computing	[154]
13	PTFE & conductive fabric	Contact separation	CNN and LSTM	90.6%	Flexible self‐powered low‐decibel voice recognition mask	[155]

**Table 5:** Applications of AI‐enhanced TENGs (contact‐separation mode) in biometric and health monitoring.

Sl. No.	Triboelectric materials	AI Techniques/ algorithms	Accuracy	Applications	Refs.
1	Ni fabric & EcoFlex	ANNs integrated FFT	98.4%	Gait analysis and patient recognition system	[48]
2	Nylon & PTFE	ML	96.6%	Real‐time sitting posture monitoring and correction vest	[161]
3	Al electrode, PTFE & ABS‐A100 frame	ML	87.17%	Smart sleep monitoring systems	[162]
4	Nitrile & EcoFlex	DL	90%	AI‐Toilet for integrated health monitoring system based on triboelectric pressure sensor array for biometrics identification	[160]
		CNN	91.15%–97.5%	AI‐Toilet for integrated health monitoring system based on commercial image sensor for urinalysis and stool analysis	
5	Cone bract leaf & PTFE	CNN	94%	Biodegradable triboelectric sensor for neck movement and body posture monitoring	[163]
6	PEDOT: PSS coated cotton & PTFE	ML	–	Self‐powered and self‐functional sock for walking‐pattern recognition and motion‐tracking	[164]
7	FEP & Cu	–	–	Self‐powered electroporation system for drug delivery	[165]
8	PET & Cu	HMI	–	Breath‐driven self‐powered sensor for monitoring real‐time human breathing	[166]
9	PA 66 & PAN	Data manipulation	–	Self‐powered E‐skin for real‐time respiratory signals monitoring and obstructive sleep apnea‐hypopnea syndrome diagnosing	[159]
10	Galinstan liquid metal & silicone rubber	CNN	98.86%, 98.4%, and 96.25%	Soft and stretchable triboelectric sensors for gait, sit and sleep monitoring	[167]
11	Nylon & PET	CNN‐BiLSTM2Attention model	97.3%	Triboelectric sensor for posture recognition, identity verification, and health monitoring	[168]
12	Nylon & PVC	RNN and GRU	94.5%	Triboelectric sensor for decoding lip language	[61]
13	Ecoflex & cardboard	RNN and LSTM	–	Double sandwich‐structured triboelectric sensor for blood pressure monitoring	[158]
14	PDMS & CNF/PVA	Data analysis	–	Self‐powered E‐skin for a smart dialing communication system and electrophysiological signals monitoring	[169]
15	Porous PDMS	DL	98.75% and 100%	Motion pattern recognition and rehabilitation monitoring	[170]

**Table 6:** Application of AI‐enhanced TENG in sports and athletics.

Sl. No.	Triboelectric materials	Mode of operation	AI techniques/ algorithms	Accuracy	Applications	Refs.
1	Wood	Contact separation	Data processing	–	Athletic big data analytics system	[180]
2	CNTs/TPE & EcoFlex	Contact separation	ML	96.7%	Self‐powered superhydrophobic triboelectric gloves for gesture recognition in VR/AR gaming	[122]
3	PET & PTFE	Sliding	Data analysis	–	Energy harvesting hybridized lower‐limb system for rehabilitation, sports monitoring, and VR applications	[175]
4	PTFE & PU	Contact separation	Data analysis	–	Wireless intelligent motion error correction system	[176]
5	PTFE & Tissue	Contact separation	K‐NN	98.1%	Dance sports and injury monitoring system and virtual game control	[177]
6	EcoFlex & Ni	Contact separation and sliding	1D CNN	97.5%	Wearable self‐powered multidimensional motion sensor VR fitness game and shooting game	[178]
7	TPU	Contact separation	Data analysis	–	Electronic skin‐based self‐powered sensing sports systems	[181]
8	PU & EcoFlex	Contact separation	Data analysis	–	Wireless intelligent sensing system for wheelchair sports monitoring	[179]

**Table 7:** Application of AI‐enhanced TENG in fault detection and optimization in industrial automation.

Sl. No.	Triboelectric materials	Mode of operation	AI techniques/ algorithms	Accuracy	Applications	Refs.
1	PET & Cu	Contact separation	SVM	83.6%	Machine fault detection system based on multimode self‐powered sensor network	[56]
2	PTFE & stainless steel	Freestanding	CNN	92.9%	Triboelectric self‐sensing bearing health monitoring	[185]
3	PTFE film & Cu	Rolling‐type freestanding	STL, AutoML model, and BO algorithm	99.48%	Triboelectric sensor‐embedded rolling bearing for the detection and recognition of rolling ball defects	[186]

#### 3.3.1 Intelligent Sensors and IoT Integration

The integration of AI and triboelectric sensors is making significant contributions to fields like the IoT, healthcare, and environmental monitoring.^[^ ^117^ ^]^ These sensors, which autonomously monitor factors, such as pressure, vibration, and temperature in real‐time, play crucial roles in predictive maintenance, health monitoring, and data‐driven decision‐making.^[^ ^118^ ^]^ One key advantage is their self‐powered nature, which eliminates the need for external power sources, enabling deployment in remote areas where traditional sensors would be impractical due to power constraints.^[^ ^119^, ^120^ ^]^ Zhu et al. demonstrated the fusion of AI, TENG, and IoT by introducing a 3D‐printed TENG device capable of converting human mechanical energy into electrical energy. This device efficiently charges a 2 µF capacitor to 1.92 V within 20 s, positioning it as a sustainable power source for small‐scale microelectronics. Additionally, this TENG‐enabled system can monitor gait metrics, including step count and frequency, using intelligent software, thereby introducing the novel concept of a “personal gait password” for secure access to personalized data. Data from this system can be wirelessly transmitted, showcasing the potential for real‐time remote monitoring. By leveraging IoT, such advancements enable interactive applications, including virtual games controlled by electrical signals.

The peak search algorithm within this system identifies key data points, enhancing the precision and effectiveness of human–computer interactions.^[^ ^124^ ^]^ AI and TENG‐based systems are at the forefront of next‐generation HMIs, improving both energy efficiency and user interface intuitiveness. These technologies combine the energy‐harvesting capabilities of TENGs with AI's data processing strength to deliver continuous health monitoring, resource optimization, and sustainable device operation. Such systems play a particularly critical role in healthcare, enabling continuous monitoring and diagnosis without relying on frequent battery replacements.^[^ ^117^ ^]^ Zhou et al., demonstrated the potential of stretchable sensors in recognizing sign language with an impressive 98.63% accuracy using Principal Component Analysis (PCA) for feature extraction and SVM for gesture recognition, achieving recognition in under one second. Integrating TENG technology into such systems would provide a reliable, sustainable power source, improving daily functionality for disabled individuals and enabling more effective communication through assistive technologies.^[^ ^125^ ^]^

**Figure 10:**
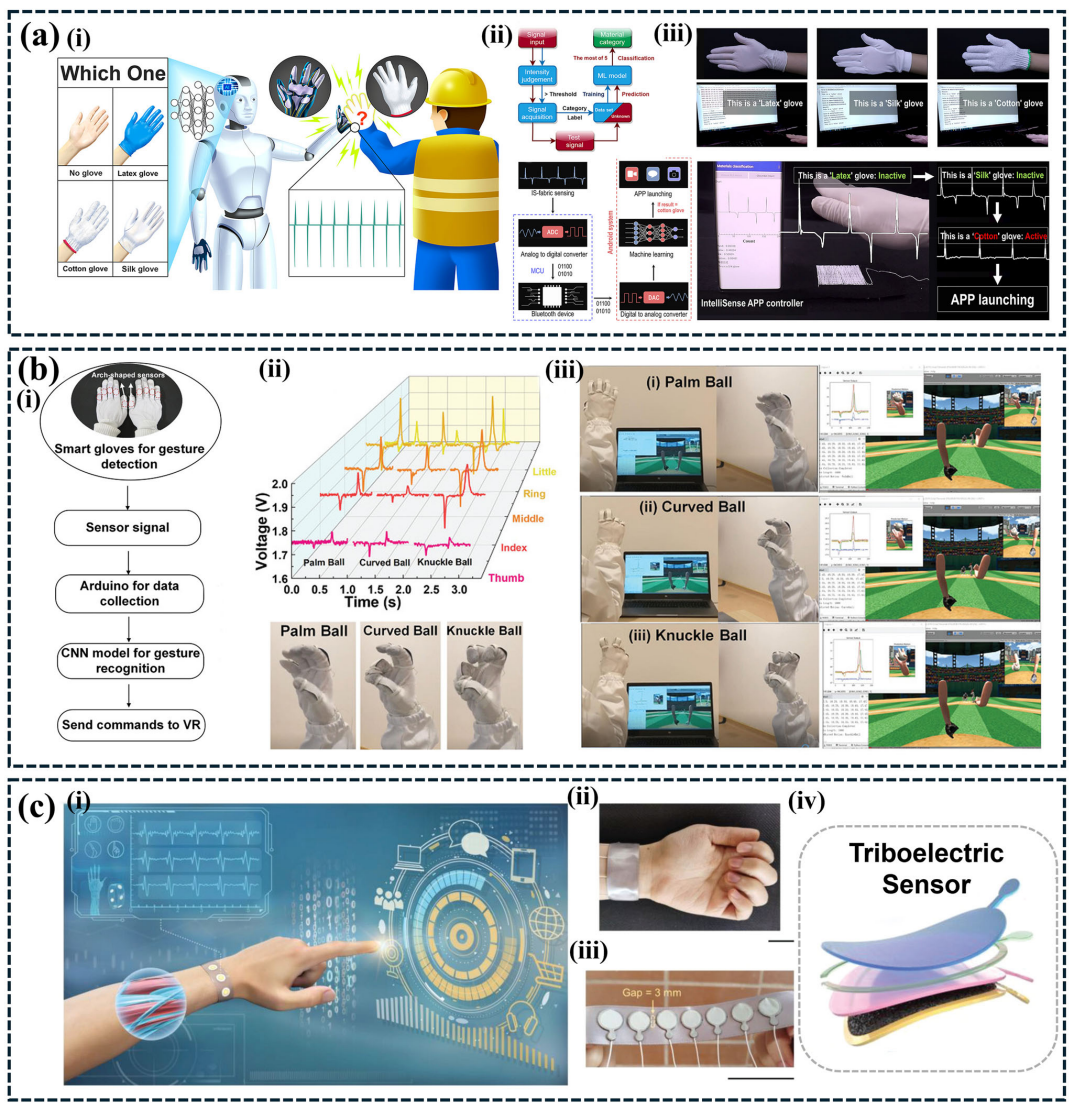
a) Material‐specific perception of IntelliSense fabrics: i) Schematic of IntelliSense fabrics for material perception and corresponding voltage variation, ii) flow chart of the signal classification process and structure of the signal classification model for material prediction, and iii) computer and Android program deployed with an ML model to distinguish different types of gloves. Reproduced with permission.^[^ ^121^ ^]^ Copyright 2022, American Chemical Society. b) Self‐powered conductive superhydrophobic triboelectric gloves for gesture recognition: i) The flow chart for gesture recognition and control, ii) signal patterns of gestures, and iii) its photograph. Reproduced with permission.^[^ ^122^ ^]^ Copyright 2020, Wiley. c) Triboelectric wristband for gesture recognition: (i) Schematic of the gesture recognition system ii, iii) photograph of triboelectric smart wristband equipped with 7 sensors, and iv) schematic structure of the triboelectric sensor. Reproduced with permission.^[^ ^123^ ^]^ Copyright 2023, Wiley.

The adoption of self‐powered sensors also generates large volumes of data, underscoring the importance of big data and AI for enhanced sensor functionality and utilization. One example is the development of artificial electronic noses, critical in detecting gas compositions. However, these sensors face challenges like gas‐sensitive drift, which can compromise measurement accuracy. AI mitigates this by analyzing sensor data in real‐time, using advanced algorithms to detect and correct for drift, thus maintaining reliable gas measurements.^[^ ^126^ ^]^ Yan et al., addressed this challenge by employing the MIDA algorithm, transforming sensor drift into a problem of distribution changes within feature space. By leveraging ML, MIDA offers a solution that stabilizes gas sensor performance over time.^[^ ^127^ ^]^ The use of AI integrated triboelectric sensor for material recognition is an innovative approach to detect and differentiate between various materials by analyzing the distinct electrical signals generated when contact with different substances having various surface textures and shapes. This capability is widely applicable for industries requiring precise material classification, such as manufacturing and quality control, as well as wearable technology that interacts with diverse surfaces. Ye et al., developed an IntelliSense core‐spun triboelectric fabric that enhances the capability of smart textiles to interact with and sense their surroundings, as shown in **Figure** **10**a. Based on the nano‐ and microscale surface roughness, the IntelliSense fabric demonstrated an ability to detect and differentiate the immediate mechanical stimuli produced by various materials. The ML model, utilizing an RNN to distinguish the signal differences with three LSTM layers to avoid misjudgments due to changes in the peak position, successfully distinguished between different materials. This technology was integrated into user‐friendly software applications for both personal computers and Android devices, facilitating real‐time predictions. With a remarkable success rate of 96.5% in identifying and classifying objects, IS fabrics demonstrate significant potential for practical applications in smart sensing and automation.^[^ ^121^ ^]^

In gesture recognition, triboelectric sensors detect minute variations in movement patterns by generating unique electrical signals for different gestures. Using triboelectric sensing gloves and wristbands, the mechanical energy from hand and wrist movements can be converted into electrical signals, allowing for real‐time gesture recognition without external power. Each gesture such as hand wave, fist clench, or finger flex, produces a distinct triboelectric output, which is processed by AI algorithms to identify and classify the motion. This functionality is especially valuable in virtual reality (VR), augmented reality (AR), and human–computer interaction, where precise gesture recognition enhances the user experience. Yen et al., developed self‐powered conductive superhydrophobic triboelectric gloves for gesture recognition in VR/AR applications, as shown in Figure 10b. Leveraging ML, the developed glove incorporates a minimalist design with one triboelectric sensor on each finger, allowing it to recognize complex and similar gestures, thereby successfully enabling advanced 3D VR/AR controls for applications, such as shooting games, baseball pitching, and floral arrangement tasks.^[^ ^122^ ^]^

In another notable development, shown in Figure 10c, Feng et al. demonstrated a passive response of triboelectric sensors to mechanical deformation from wrist tendons and muscles for the identification of sophisticated hand movements with AAL.^[^ ^123^ ^]^ Furthermore, in healthcare, triboelectric gloves and wristbands can assist in rehabilitation by tracking patients' hand and wrist movements, providing essential data for tailored physical therapy programs. They also hold promise for assistive technology, enabling those with disabilities to interact with their environment via gestures. Triboelectric gloves can be programmed to translate sign language into audible speech, facilitating communication for the hearing‐impaired. The integration of AI improves the accuracy of these devices, making them increasingly adaptable for various applications. Tang et al. introduced an innovative nontouch gesture recognition device, developed with monolayer and polyethylene terephthalate substrates. This transparent, lightweight, and flexible sensor adheres easily to display surfaces, suggesting future applications where screens themselves generate power. This advance not only facilitates touchless control for applications, such as medical robotics but also opens possibilities in augmented and virtual reality systems.^[^ ^140^ ^]^ Self‐powered sensors equipped with AI capabilities offer sustainable energy solutions by harnessing mechanical, thermal, or environmental energy, reducing dependence on batteries. The combination of AI and TENG technology significantly enhances sensor functionality, enabling real‐time data analysis, adaptive responses, and pattern recognition. These advancements hold far‐reaching implications in various domains, from continuous healthcare monitoring to efficient smart city resource management, ultimately contributing to sustainability, enhanced decision‐making, and improved HMIs.^[^ ^18^ ^]^

#### 3.3.2 HMIs and Robotics

TENG technology enhanced by AI, represents a novel approach to improving HMI applications. When AI‐based TENGs are integrated into HMIs, they allow touch and gesture recognition capabilities that are not just responsive but also energy‐efficient.^[^ ^141^ ^]^ This allows users to engage with devices and interfaces through touch, swipes, and other gestures, while the AI component interprets these inputs to enhance user experiences and operational efficiency. As self‐powered devices, TENG‐based HMIs contribute to sustainability and environmental friendliness, making them an intriguing technology for the future of interactive systems.^[^ ^142^, ^143^ ^]^ Ye et al., developed an integrated device with a dual‐mode temperature‐regulating function inspired by penguins and a self‐powered triboelectric HMI, with the goal of improving human–machine interaction in difficult circumstances. This TENG system emulates electronically coded Morse code, thereby revolutionizing methods for information encryption, lamp signal transmission, and emergency calls in previously uncharted areas. Utilizing electrospun cooling and heating materials with photothermal conversion capabilities, it provides personal thermal control. When pressure is applied, the TENG generates innovative interaction techniques due to its exceptional electrical output capabilities. Users can create signal sequences by applying pressure with their fingertips, supported by a signal acquisition circuit that facilitates the generation of Morse code. To achieve high recognition accuracy, ML approaches record these signals 60 times and use four different algorithms, including RF, ET, DT, and SVM. Notably, the DT and SVM algorithms attain higher levels of recognition accuracy, with average recognition accuracy ranging from 96% to 100%. By merging novel technology with ML for improved signal detection and interaction, this work reveals the possibility of advanced human–machine interaction.^[^ ^144^ ^]^ Similarly, Yuan et al., developed a contactless NTENG that combines graphene with a shear‐strengthening elastomer to enhance self‐charging, shock resistance, and self‐healing properties.

This NTENG, which features a graphene electrode and a specialized shell, monitors the distance and speed of moving objects autonomously while also dissipating 41.6% of the impact force through the combined effect of electrostatic induction and triboelectric phenomena. These self‐healing capabilities of NTENG components can be used to create a variety of 3D structures, such as cubic and triangular arrays. This technology was applied to a walking stick in an HMI setting, where three NTENG units were combined to create a linear sensor array. This setup enabled users to determine directions (left, forward, and right) and navigate in low‐light conditions through electric impulses. Additionally, the NTENG array served as a self‐powered wearable electronic system for noncontact human motion tracking, with luminescent LEDs visually displaying fingertip trajectories, offering valuable insights for HMI applications.^[^ ^145^ ^]^ Akshaya and Stylios recently developed a comprehensive IoT‐enabled touch sensor system using CuNi‐BEF EcoFlex layered F‐TENGs, capable of accurately detecting both soft and hard touches. This advanced system enhances robotic functionality by enabling nuanced touch sensitivity, essential for precision tasks, and fostering more intuitive human–machine interactions.^[^ ^146^ ^]^

**Figure 11:**
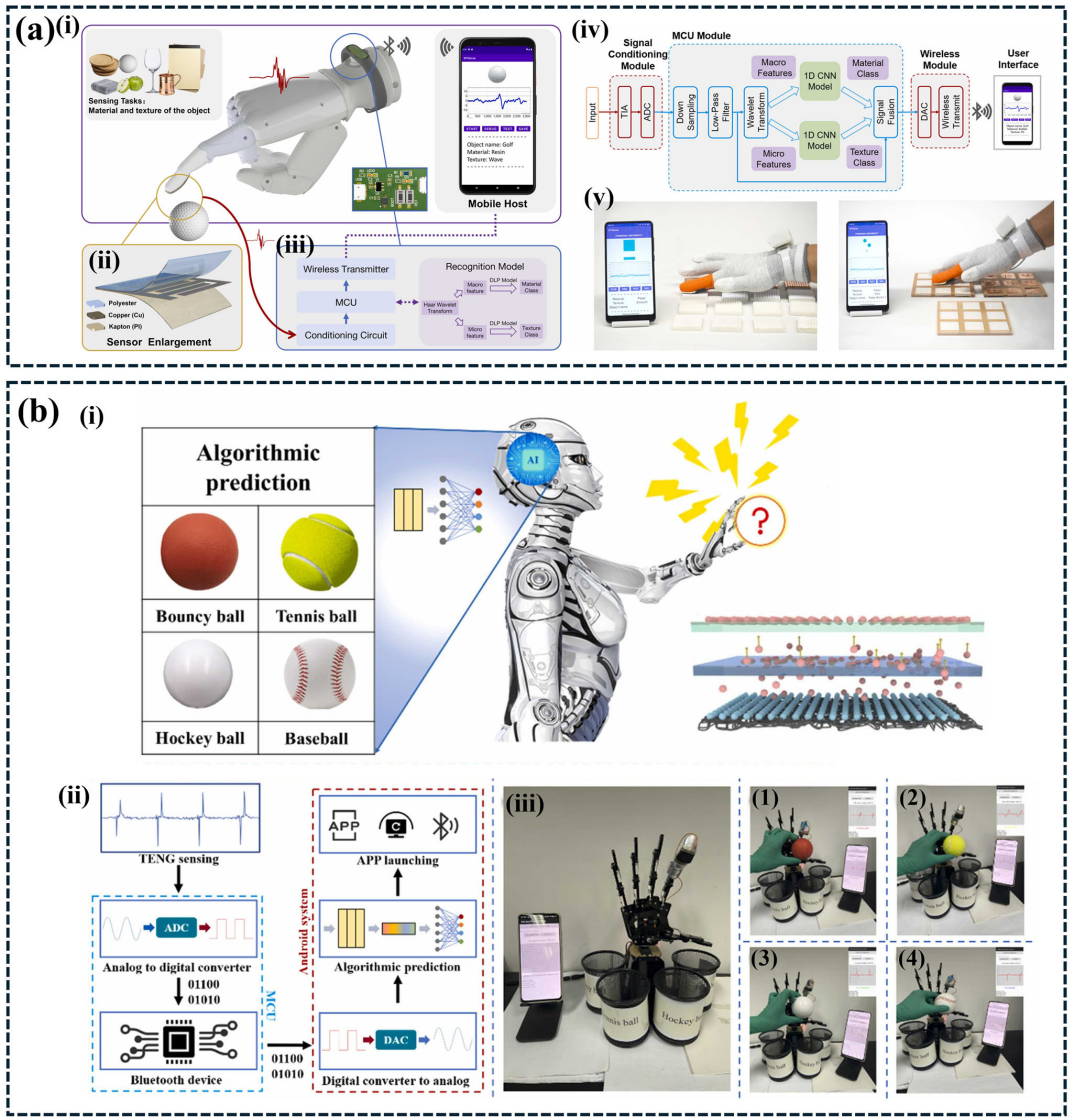
a) Flexible triboelectric tactile sensing system for material and texture recognition: i) Schematic workflow of the tactile sensing system, ii) flexible fingertip freestanding mode triboelectric sensor, iii) system‐level block diagram of the data acquisition and recognition processing, iv) Working mechanism of classical signal processing and DL algorithm of the sensing system, and v) Real time simultaneous material and texture recognition. Reproduced with permission.^[^ ^128^ ^]^ Copyright 2022, Elsevier. b) Ball intelligent perception sorting: i) Schematic illustration for different ball perception domains, and the structure of the TENG device, ii) signal processing process of Android application, iii) incorporation of TENG with robot and Android application to identify and sort different balls 1) bouncing ball, 2) tennis ball, 3) hockey ball, and 4) baseball. Reproduced with permission.^[^ ^28^ ^]^ Copyright 2023, Elsevier.

Electronic skin equipped with tactile perception has empowered smart robots and prosthetics to execute complex manipulation tasks and interact effortlessly with humans and their environments. Drawing inspiration from the sensory capabilities of human fingertips, Song et. al. developed a wireless integrated tactile sensing system that can simultaneously recognize materials and textures based on grating‐structural freestanding TENG‐based electronic skin sensors (**Figure** **11**a). Subsequently, this sensor can predict the materials and textures of the contacted objects with accuracies of 99.07% and 99.32%, respectively, deploying DL techniques.^[^ ^128^ ^]^ A single electrode mode TENG integrated with an intelligent sensing system that can control and monitor electronic and electrical systems, is shown in Figure 11b. This system can detect and differentiate the mechanical contacts produced by balls made from various materials, achieving a high prediction accuracy of 96.8% with the help of a DL method combining CNN and GRU.^[^ ^28^ ^]^

#### 3.3.3 Healthcare Applications

AI‐based TENG technology is critical in health and biological applications, enabling continuous health monitoring through wearable devices that provide real‐time data for managing chronic illnesses and emergencies.^[^ ^156^ ^]^ The data analytics and predictive powers of AI facilitate the processing of the massive amounts of health data collected by TENG sensors, allowing for early disease detection and personalized risk assessment.^[^ ^157^ ^]^

This technology also enhances the efficiency and responsiveness of assistive devices, drug delivery systems, and diagnostic instruments which is particularly beneficial for patients with chronic conditions who require constant monitoring to prevent complications and hospitalizations. Furthermore, the combination of TENG with AI supports the development of smart implants, which improves patient care and the adaptability of medical equipment. Overall, AI‐powered TENG technology is driving transformative advances in healthcare, enhancing patient outcomes and medical efficiency.^[^ ^115^ ^]^ Jiang et al. introduced a self‐powered sitting position monitoring vest (SPMV) that harnessed the energy from user movements through a double weft‐knitted fabric containing nylon yarns and conductive fibers, ensuring both comfort and stretchability. Sensors integrated into the vest's fabric capture real‐time data on body part deformations, improving position recognition accuracy. ML algorithms process the signals, enabling precise identification of the user's posture. The SPMV achieved a remarkable location identification accuracy of 96.6% using a RF classifier, surpassing logistic regression and DT classifiers. This TENG‐based SPMV represents a promising application of triboelectric wearable technology, offering a reliable solution for posture adjustments during sitting or resting activities.^[^ ^161^ ^]^

A continuous real‐time blood pressure monitoring system, developed by Ran et al., incorporates a double sandwich‐structured triboelectric sensor and employs a DL algorithm. The system achieved an accuracy of 3.75 ± 5.27 mmHg for systolic and 3.86 ± 5.18 mmHg for diastolic blood pressure. This system provides a sustainable, self‐powered method for estimating blood pressure, highlighting the successful integration of AI and wearable electronics in healthcare applications.^[^ ^158^ ^]^

**Figure 12:**
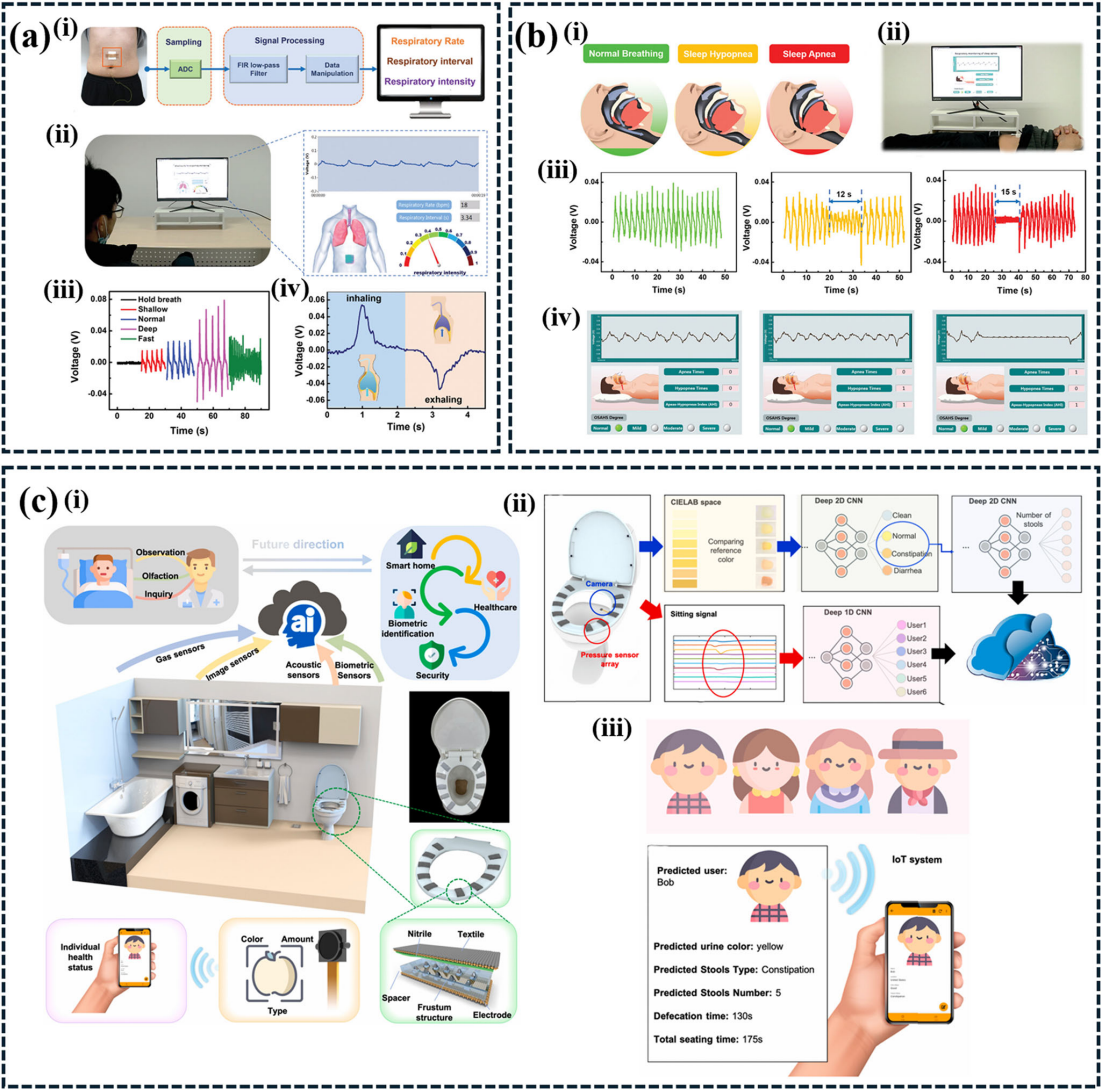
a) Respiratory monitoring system: i) Flow diagram of data acquisition and processing for respiratory monitoring, ii) interface of the real‐time respiratory monitoring system, iii) real‐time voltage signals of different respiratory states, and iv) respiratory waveform of inhaling and exhaling. b) Obstructive sleep apnea‐hypopnea syndrome diagnosis system: i) The respiratory airway states while asleep, ii) photograph of obstructive sleep apnea‐hypopnea syndrome diagnosing, iii) real‐time voltage signals under different sleep respiratory states, and iv) sleep respiratory state analysis based on the voltage signals. Reproduced with permission.^[^ ^159^ ^]^ Copyright 2021, Wiley. c) AI‐Toilet: i) Schematic illustration of AI‐Toilet using triboelectric pressure sensors and image sensor for integrated health monitoring system, ii) working mechanism of integrated health monitoring system, and iii) mobile application showing the health information. Reproduced with permission.^[^ ^160^ ^]^ Copyright 2021, Elsevier.

Peng et al. introduced a high sensitivity, self‐powered breathable electronic skin‐based TENG for real‐time respiratory monitoring and obstructive sleep apnea‐hypopnea syndrome (OSAHS) diagnosis. A real‐time respiratory monitoring and sleep breathing detecting system as displayed in **Figure** **12**b,c, respectively, improves sleep quality and thereby delays or prevents the risk of OSAHS development.^[^ ^159^ ^]^ Liu et al. developed an ML‐driven smart motion and rehabilitation monitoring system (SMRMS) for full‐body motion recognition and rehabilitation using a porous PDMS‐based TENG array. The porous triboelectric sensor achieved a sensitivity of 1.76 kPa^−1^ and a fast recovery time of 50 ms. A ring‐shaped TENG was fabricated to enable real‐time leg and arm force monitoring by integrating with a multichannel signal acquisition system. The collected motion data were analyzed using ML, achieving 98.75% accuracy in motion pattern recognition and 100% accuracy in rehabilitation monitoring. These advanced, self‐powered, and highly adaptable sensors enhance wearable technology for injury prevention and personalized rehabilitation tracking.^[^ ^170^ ^]^ An AI‐powered smart toilet has been developed for integrated health monitoring, incorporating a textile‐based triboelectric pressure sensor array for biometric identification and a commercial image sensor for urinalysis and stool analysis (Figure 12d)). The system can detect seating pressure up to 200 kPa, while DL algorithms achieve over 90% accuracy in identifying six users. The IoT module's image sensor tracks color variations for urinalysis, and CNNs classify stool types with 97% accuracy and stool amounts with 91%. All collected health data are uploaded to a server for continuous monitoring and displayed on users' mobile devices.^[^ ^160^ ^]^ The data analytics and prediction capabilities of AI provide significant insights that aid in early disease identification and personalized therapy. Furthermore, AI‐based TENG technology aids in the creation of smart implants, rehabilitation, and the advancement of improved diagnostic tools, ultimately enhancing patient outcomes and healthcare efficiency.^[^ ^171^ ^]^ This integration transforms the healthcare landscape by enabling better‐informed decision‐making, personalized therapies, and improved patient care.^[^ ^172^ ^]^

#### 3.3.4 Sports and Athletics

**Figure 13:**
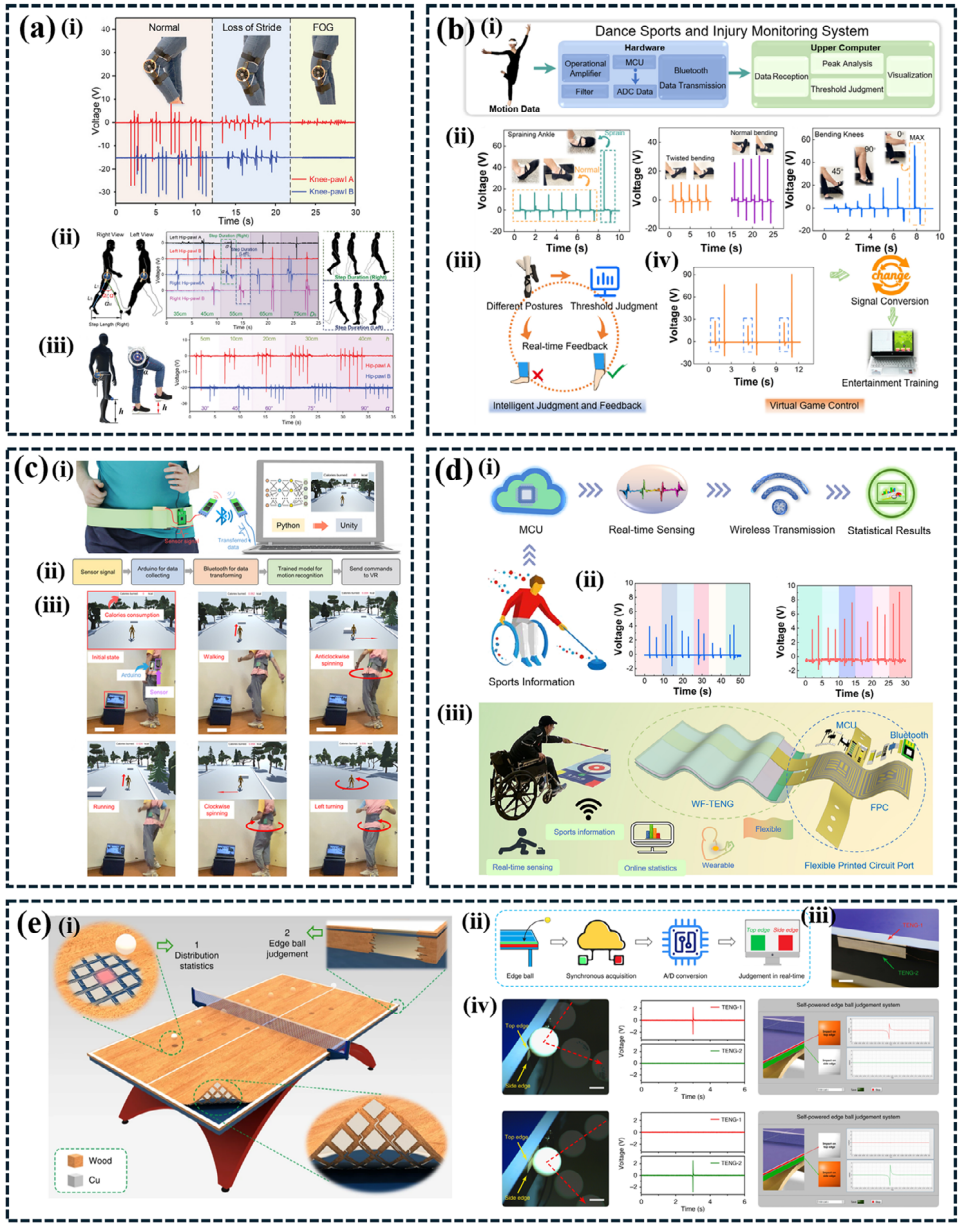
a) Lower‐limb motion monitoring with TENGs for rehabilitation applications: i) The detection of rotation angles through the TENG on the knee joint to monitor typical parkinsonian gaits, voltage signals of the TENG corresponds: ii) to different step lengths in a full gait cycle, and iii) on the hip with different heights of foot. Reproduced with permission.^[^ ^175^ ^]^ Copyright 2021, Wiley. b) Dance sports and injury monitoring system: i) Schematic diagram, ii) monitoring ankle sprains, normal flexion and extension, and postinjury flexion and extension of the ankle joint and knee hyperextension, and the flow diagram for iii) judging dance sports status and iv) virtual game control. Reproduced with permission.^[^ ^177^ ^]^ Copyright 2024, Elseiver. c) VR fitness game based on the multidimensional motion sensor: i) Schematic diagram, ii) the flowchart for the control system and process, and iii) demonstrations of virtual character controlling. Reproduced with permission.^[^ ^178^ ^]^ Copyright 2023, Science Partner Journal. d) Wearable wireless intelligent triboelectric sensing system (WISS) for wheelchair sports monitoring: i) Schematic diagram of WISS curling ii) the generated output voltage signals, and iii) schematic of wearable flexible (WF) TENG sensing port structure design. Reproduced with permission.^[^ ^179^ ^]^ Copyright 2023, Elsevier. e,i) Schematic representation of the natural wood‐based smart ping‐pong table, ii) working mechanism of TENG‐based self‐powered edge ball judgment system, iii) photograph of two TENGs attached on the edge of the ping‐pong table, and iv) demonstration of the real‐time self‐powered edge ball judgment system. Reproduced with permission.^[^ ^180^ ^]^ Copyright 2019, Nature.

AI‐based TENG technology delivers self‐powered and responsive solutions in sports and wearables applications, allowing for continuous data monitoring and analysis. Athletes can benefit from smart clothing embedded with TENG sensors that monitor a variety of performance metrics, such as heart rate, movement patterns, and body posture.^[^ ^173^ ^]^ It improves the performance and functionality of wearable devices by providing athletes and individuals with real‐time feedback, energy‐efficient operation, and improved motion detection. Additionally, this self‐sustaining feature improves convenience and ensures that athletes benefit from uninterrupted monitoring throughout training sessions or competitions.^[^ ^49^, ^174^ ^]^ The data collected by these smart wearables can be analyzed using AI algorithms to provide athletes with actionable insights. AI can help athletes understand their optimal training loads, suggesting adjustments based on real‐time performance data to avoid overtraining or injury. By providing personalized recommendations, athletes can tailor their training regimens to maximize performance while minimizing the risk of injury.^[^ ^171^ ^]^ Gao et al. introduced a hybrid energy harvesting and motion‐capturing lower‐limb system for rehabilitation and sports applications. To demonstrate the ability of the system for real‐time lower‐limb motion sensing in clinical diagnosis, it is attached to the knee joint to measure three imitated gait features, such as slightly abnormal, highly abnormal, and reduced strength. The variations in knee bending angles were recorded, with larger, irregular angles seen in highly abnormal gait, while smaller, more regular angles indicated reduced strength. Additionally, the triboelectric lower‐limb motion sensing system successfully detected freezing of gait in a Parkinsonian gait, highlighting its potential to prevent falls by identifying sudden mobility loss. **Figure** **13**a shows the lower‐limb posture recognition by detecting the rotation angle and direction of the lower‐limb joints, providing a feasible solution in rehabilitation as well as healthcare monitoring applications.^[^ ^175^ ^]^

TENG‐based wireless intelligent motion correction system that tracks fitness activities and facilitates movement error correction by integrating with an intelligent host computer system for signal processing and visualization. By continuously harvesting mechanical energy from human motion, the system evaluates movement parameters, aiding users in developing proper techniques and reducing injury risks from improper movements.^[^ ^176^ ^]^ The dance sports injury monitoring system (DIMS), incorporating triboelectric sensors on the knee and ankle joints, effectively monitors dancer movements, including jumping techniques and injury risks. The system tracks voltage changes corresponding to joint flexion, extension, and stability, providing real‐time data on movement and injury prediction, such as ankle sprains or knee hyperextension, as shown in Figure 13b. This information is wirelessly transmitted for analysis, with feedback displayed in animations to aid training and rehabilitation. Additionally, DIMS integrates virtual game controls to enhance engagement and training efficiency in dance practice.^[^ ^177^ ^]^ Guo et al. developed an indoor wearable intelligence sport system that utilizes a multidimensional motion sensor integrated into a belt to accurately capture motion signals from the waist during various activities, including walking, running, and turning, as shown in Figure 13c. The system employs an SVM model capable of recognition accuracy of up to 93.8% after training on a dataset collected over multiple days. They also implemented a virtual reality fitness game that translates these movements into corresponding actions for a virtual character, allowing for real‐time interaction and calorie tracking during exercise. The recognition accuracy of 95% achieved for distinguishing waist‐spinning velocities has been further enhanced using a one‐dimensional CNN for recognizing more complex shank motions, resulting in an impressive accuracy of 97.5%. This system showcases the potential of AI‐driven wearable technology in enhancing sports training and interactive fitness experiences.^[^ ^178^ ^]^ Furthermore, TENG‐enabled devices can facilitate motion capture and analysis in sports. By using multiple sensors strategically placed on an athlete's body, coaches, and trainers can gain a comprehensive understanding of their movements. This information can be invaluable for sports, such as baseball, basketball, or gymnastics, where precise motion is crucial for success. Wen et al. introduced an innovative application of CNN with a TENG sensor glove designed for the recognition of baseball throwing gestures. The glove collected and transmitted gesture data through TENG sensors embedded within it. The CNN, in turn, analyzed these electrical signals to accurately identify various throwing gestures, including the palm ball, curved ball, and knuckleball. The trained CNN demonstrated an impressive gesture recognition accuracy of 99.167%. This technology has broader implications in virtual sports, where it can capture athlete motion data through sensors, enabling the simulation of real‐game scenarios and enhancing training and performance analysis in sports.^[^ ^122^ ^]^ Shi et al. developed a flexible, breathable, and antibacterial TENG‐based E‐skin with a nanofiber network and a 3D layered porous structure is developed for self‐powered sensing in volleyball reception statistics and analytics.

The self‐powered wearable E‐skin sensor array is adopted to volleyball is capable the recording real‐time training data, providing reasonable training evaluation for athletes and competition tactics arrangement for the coach.^[^ ^181^ ^]^ Figure 13d illustrates a wearable wireless intelligent triboelectric sensing system (WISS) for technical judgment and analysis on wheelchair curling sports. The TENG sensors attached to the elbow joint monitor the throwing technique in wheelchair curling by converting motion signals into electronic data for real‐time analysis by capturing elbow flexion angles and output voltage during different throwing techniques, aiding in performance evaluation. WISS provides real‐time feedback through animations, offering athletes, and coaches valuable insights for technique refinement and training optimization, demonstrating its the potential of wearable electronics in enhancing sports performance and injury prevention in adaptive sports.^[^ ^179^ ^]^ Luo et al., proposed wood‐based TENG for self‐powered sensing in athletic big data analytics and edge ball judgment system, as shown in Figure 13e. The TENG is integrated into a smart ping‐pong table with self‐powered systems for real‐time training data collection and edge ball judgment, offering new opportunities for sustainable sports technologies and data‐driven athletic performance analysis.^[^ ^180^ ^]^

#### 3.3.5 Industrial Automation

The convergence of AI and TENGs is revolutionizing the landscape of industrial automation. AI, with its ability to learn, reason, and adapt, offers advanced control and optimization of industrial processes. TENGs, on the other hand, provide a sustainable, self‐powered solution for energy harvesting, enabling autonomous operation of AI‐driven systems without external power sources.^[^ ^182^ ^]^ AI has been deployed in various aspects of industrial automation, including predictive maintenance, quality control, process optimization, and autonomous robotics. By analyzing sensor data, AI algorithms can predict equipment failures, reducing downtime and maintenance costs. Those AI‐powered TENG systems can inspect products for defects, ensuring high‐quality output.^[^ ^183^ ^]^ At the same time, AI can also optimize production processes by identifying bottlenecks and inefficiencies, leading to increased productivity and reduced costs. Additionally, AI enables robots to perform complex tasks autonomously, increasing safety and efficiency. TENGs complements AI by providing a sustainable and self‐powered solution for industrial automation. They can power sensors used to monitor equipment health, environmental conditions, and product quality, as well as actuators for controlling machinery and equipment, reducing the reliance on external power supplies. Moreover, TENGs can generate energy to power AI‐driven devices, allowing autonomous operations independent of traditional power grids.^[^ ^184^ ^]^

**Figure 14:**
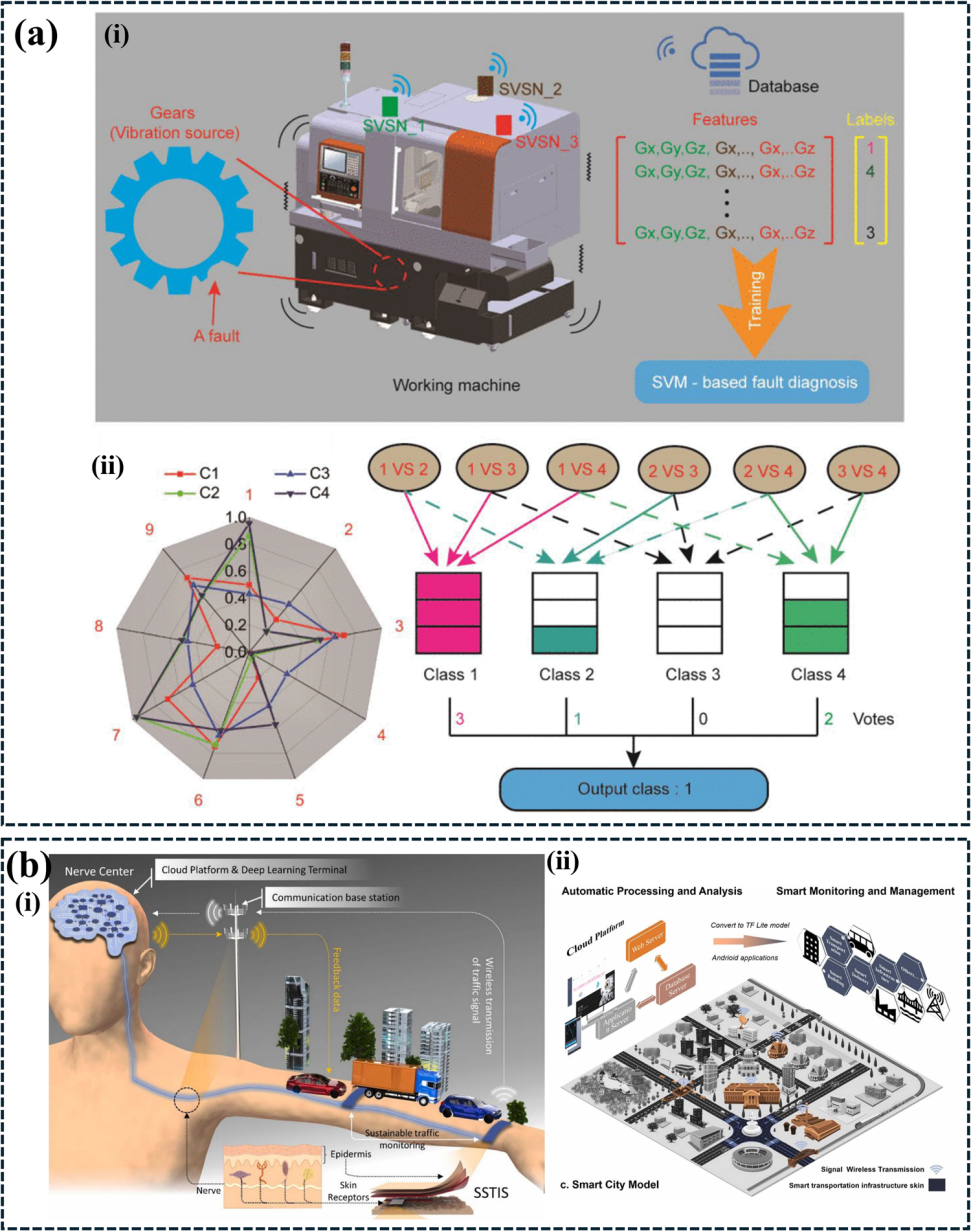
a) Machine fault detection based on multimode self‐powered sensor network: i) Schematic representation of the machine fault detection system, and ii) SVM‐based fault detection. Reproduced with permission.^[^ ^56^ ^]^ Copyright 2020, IEEE. b) Self‐powered smart clean energy‐based transportation infrastructure skin system: i) Schematics of a smart transportation infrastructure skin system in smart cities, and ii) working mechanism of sensing, wireless transmission, and analysis of signal data of transportation infrastructure in the smart cities using smart transportation infrastructure skin system. Reproduced with permission.^[^ ^190^ ^]^ Copyright 2022, Elsevier.

A prime example of AI and TENG integration is in AI‐driven predictive maintenance. TENG‐powered sensors can monitor equipment vibration and temperature, collecting data continuously. AI algorithms analyze this data to detect anomalies that indicate potential equipment failures. By predicting failures in advance, industries can schedule maintenance proactively, reducing downtime and improving overall efficiency. Li et al., developed a self‐powered multinode sensor network using a multilayer vibrational TENG to harvest vibration energy from machines, to establish a machine fault detection system, as shown in **Figure** **14**a. This TENG‐based sensor network achieved an accuracy of 83.6% in machine fault detection, highlighting its potential for applications in production monitoring, intelligent manufacturing, and smart factories.^[^ ^56^ ^]^ Furthermore, enhanced product quality and safety could be achieved through AI‐based triboelectric sensor systems. Ultimately, the integration of AI and TENGs has the potential to transform industries into more sustainable, efficient, and autonomous operations.

#### 3.3.6 Smart Cities and Infrastructure

The integration of AI and TENG technology in smart cities has the potential to transform urban life and resource management. TENGs, which harvest energy from mechanical motions like vibrations, wind, and human movement, can be embedded into urban infrastructure, such as roads, buildings, and public spaces to capture energy from everyday activities.^[^ ^187^ ^]^ This energy can power low‐consumption devices like IoT sensors and streetlights, while AI ensures efficient energy distribution through demand forecasting, storage management, and load balancing within the city grid.^[^ ^18^ ^]^ TENGs also serve as self‐powered sensors in infrastructure, detecting structural issues, such as cracks or vibrations in roads and buildings. AI can analyze this data to predict maintenance needs, prevent failures, and reduce costs.^[^ ^184^ ^]^ For example, TENG devices installed along roadways can harness energy from vehicles and pedestrian movements, which could power electric vehicle charging stations or traffic systems. AI can optimize traffic flow with real‐time data, reducing congestion and energy consumption. Additionally, AI can automate electric vehicle charging cycles powered by TENG energy, enhancing urban transport efficiency.^[^ ^188^, ^189^ ^]^

In smart buildings, TENGs can capture energy from vibrations and human movement, while AI manages power usage based on occupancy patterns and environmental conditions.^[^ ^191^ ^]^ For environmental monitoring, TENG‐powered sensors can track air quality, with AI analyzing data to predict adjustments that reduce pollution and improve living conditions.^[^ ^189^ ^]^ Furthermore, TENGs can power AI‐driven surveillance systems, ensuring public safety in remote or energy‐limited areas by analyzing video feeds in real time. During natural disasters, AI‐coordinated emergency responses using real‐time data from TENG‐powered sensors embedded in infrastructure could enhance disaster management. This synergy between AI and TENGs supports sustainable and resilient urban planning, fostering energy‐efficient and environmentally friendly smart cities.^[^ ^192^ ^]^ Together, these technologies promise to create adaptive, self‐sustaining cities, paving the way for future urbanization. Beyond energy efficiency, their combined capabilities extend to waste management and water system optimization. TENG‐powered sensors can be placed on trash bins and collection vehicles to monitor waste levels, while AI analyzes the data to optimize waste collection routes, reducing fuel consumption and improving efficiency.^[^ ^188^ ^]^

In water management, TENG sensors can monitor water flow, leakages, and quality while generating energy from the movement of water through pipelines. AI would analyze this data to detect inefficiencies, predict equipment failures, and optimize water distribution, conserving resources and reducing costs, particularly in water‐scarce regions.^[^ ^192^ ^]^ AI and TENG technologies can also enhance civic engagement and urban decision‐making. TENG‐powered devices, such as interactive public kiosks and smart street furniture, can collect data on citizen behavior and preferences. Data analysis by AI can help to improve public services, transportation schedules, and even the design of public spaces.^[^ ^187^ ^]^ Another crucial application of AI and TENG technology is in climate resilience. Cities facing challenges from climate change, such as flooding, heatwaves, and storms, could benefit from TENG‐powered environmental sensors that collect data on weather conditions and structural integrity in vulnerable areas.^[^ ^193^ ^]^ AI can use this data to model disaster scenarios, optimize emergency response, and better prepare cities for extreme weather events. Additionally, the integration of 5G and AI‐enhanced IoT devices, powered by TENG‐generated energy, will further enhance smart cities by creating hyper‐connected environments where urban systems are optimized for efficiency, sustainability, and resilience.^[^ ^184^ ^]^ This synergy between AI and TENG technology offers smart cities a sustainable, efficient, and adaptive approach to urban challenges, by not only addressing current issues but also equipping cities to meet future needs and uncertainties, paving the way for intelligent, self‐sustaining urban environments.^[^ ^18^ ^]^

Zheng et al., proposed a self‐powered smart transportation infrastructure skin (SSTIS) system for the precise monitoring of various civil infrastructures, such as roads, vehicles, roads, buildings, and bridges, ensuring smooth and efficient operation within a smart city, as illustrated in Figure 14b. By leveraging TENG technology, this system harvests energy from everyday movements and vibrations, providing real‐time data for the management and maintenance of urban infrastructure. Additionally, using TensorFlow Lite by Google, mobile intelligent monitoring and analysis and an Android application called “Smart Road” has been developed. This app can be deployed on smartphones and autonomous vehicles, facilitating real‐time computation for traffic monitoring and infrastructure interaction. Through wireless communication technology, “Smart Road” ensures fast and reliable interactions between road users and traffic systems. By combining AI and TENG technology, SSTIS offers a self‐powered, real‐time, and sustainable solution for monitoring and managing smart city infrastructure, contributing to the intelligent, adaptive, and energy‐efficient urban environments of the future.^[^ ^190^ ^]^

## 4 Challenges and Future Scope for AI‐Driven TENG Innovations

The integration of AI‐driven TENG systems holds transformative potential for advancing intelligent, self‐powered devices. This synergy enables a comprehensive pathway from material optimization to be advanced, real‐time sensing applications. By leveraging ML and DL algorithms, AI can enhance TENG‐based systems, improving energy harvesting, predictive maintenance, and real‐time adaptability, which allows these systems to perform reliably even in complex, dynamic environments. This approach paves the way for groundbreaking applications, including implantable technologies, such as brain–computer interfaces (BCIs)‐ Elon Musk's Neuralink,^[^ ^202^ ^]^ autonomous environmental monitoring sensors,^[^ ^25^, ^203^ ^]^ wearable health diagnostics,^[^ ^204^, ^205^ ^]^ and smart infrastructure.^[^ ^49^, ^206^ ^]^ These applications form robust networks of self‐powered IoT devices, capable of high‐precision data acquisition and responsive analysis across diverse fields. Despite these promising advancements, several critical technical and operational challenges remain to be addressed to unlock the full potential of AI‐driven TENG technologies.

Current research focuses on tackling these challenges, which include enhancing data processing and real‐time performance to ensure responsiveness in high‐demand scenarios and optimizing TENG layer structure and device architecture to improve energy conversion and integration. Ensuring high accuracy and consistency in data collection remains essential for reliable AI‐driven predictions while developing universal predictive models for TENG performance will expand their adaptability across different applications. Moreover, advancing AI algorithms specifically tailored for TENG optimization is crucial for maximizing efficiency and adaptability. Sustainability is also a key priority, with research into novel materials that are both high‐performing and environmentally friendly.

**Figure 15:**
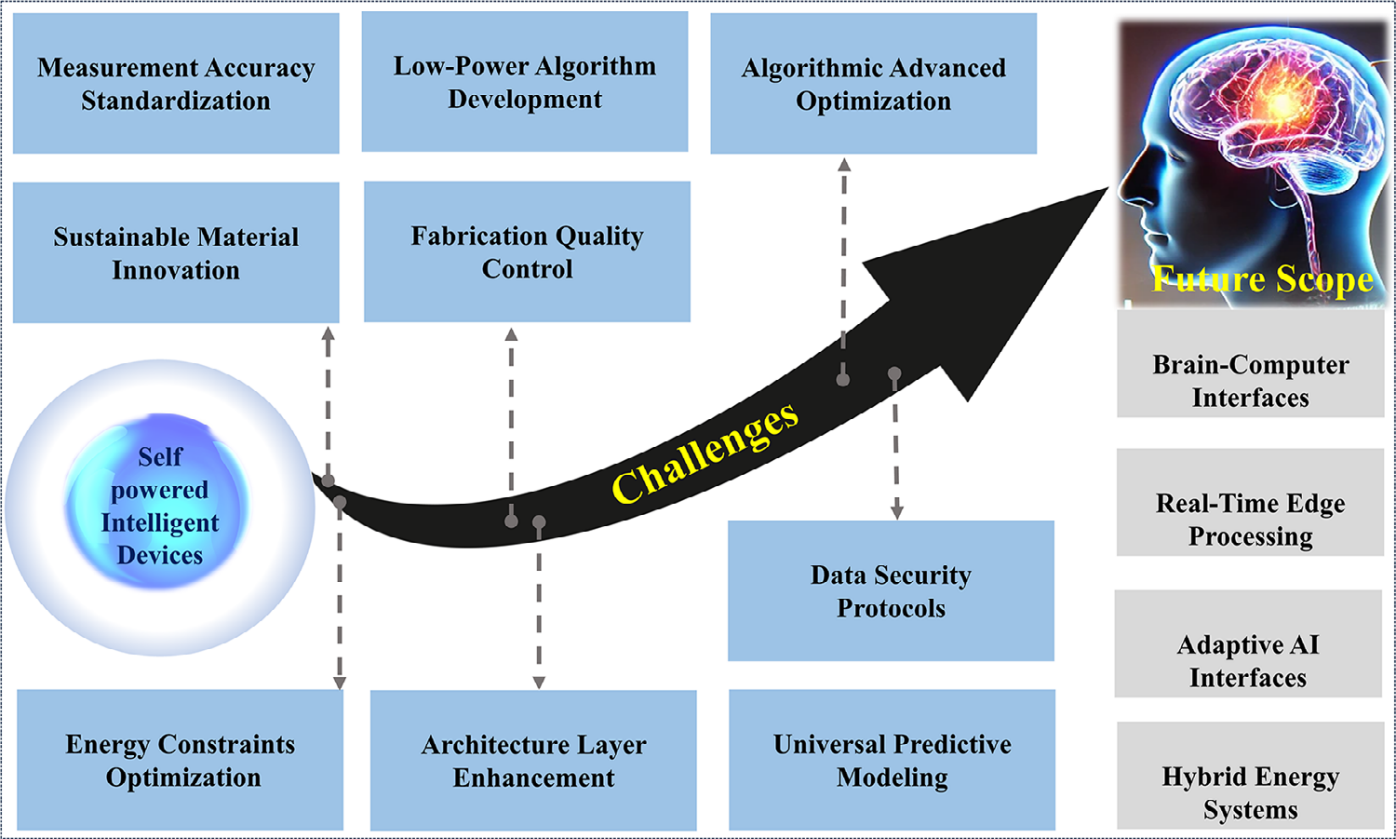
Schematic overview of current research challenges and future directions for AI‐driven TENGs.

To ensure high standards, AI‐powered fabrication and quality control are being explored to improve precision and standardization across TENG devices. Addressing measurement and reporting consistency gaps in AI‐powered TENG systems will also be critical for performance evaluation. To make these systems fully self‐sustaining, low‐power AI algorithms are being developed, minimizing the energy needed for continuous operation. Hybrid TENG solutions that combine energy sources are also under investigation to create multisource harvesting systems capable of capturing power from diverse environmental inputs. Finally, robust security protocols are essential to protect data and maintain the integrity of AI‐enhanced TENG applications. By addressing these challenges, we can set the stage for the next generation of AI‐enhanced TENG applications, encompassing innovations in low‐power AI, sustainable materials, and hybrid energy systems. This will pave the way for a future of intelligent, sustainable, and self‐powered devices that serve critical roles across various sectors. **Figure** **15** illustrates a schematic overview of current research challenges and future directions for AI‐driven TENGs.

### 4.1 Data Processing and Real‐Time Performance Analysis

AI‐driven TENG systems rely heavily on efficient data processing to achieve real‐time adaptability, anomaly detection, and predictive maintenance. These tasks, essential for autonomous operations, typically utilize advanced ML and DL algorithms. However, the high computational demands of these algorithms present challenges, as they consume considerable energy, impacting the self‐powered efficiency of TENGs. For TENG systems to operate effectively, lightweight AI algorithms tailored to energy constraints are critical. To address this, research has turned toward Edge AI strategies, such as federated learning and TinyML, which allow data processing at the device level rather than depending on cloud infrastructure. This local processing approach conserves energy by reducing data transmission needs and lowering latency, making TENGs more sustainable for real‐time applications. For instance, models like MobileNet and TinyML have demonstrated the potential to achieve classification accuracies of up to 85% in real‐time applications while operating on minimal power, a significant improvement for self‐powered IoT devices. Developing and optimizing these models specifically for TENG hardware could further enhance efficiency, making TENGs more feasible for continuous intelligent operation in areas such as IoT and healthcare. In IoT, TENG devices equipped with on‐device intelligence could autonomously monitor and adjust environmental conditions or track device wear, offering predictive maintenance insights with reduced energy costs.^[^ ^107^, ^207^ ^]^ In healthcare applications, wearable TENGs could potentially monitor vital signs, detect abnormal patterns, and alert healthcare providers in real time with minimal external energy requirements.^[^ ^19^, ^94^ ^]^

### 4.2 TENG Layer Structure and Device Architecture

The structure and material composition of TENG layers—electrodes, triboelectric surfaces, spacers, and substrates—are crucial for optimizing energy output, stability, and durability. Each layer uniquely affects energy conversion efficiency, with performance influenced by the interplay of tribomaterials properties like electron affinity, dielectric constant, elasticity, and mechanical strength, alongside the mechanical dynamics driving charge generation. While methods, such as constrained particle swarm optimization have been applied to specific TENG structures,^[^ ^204^, ^205^ ^]^ their utility is limited to fixed configurations, restricting broader applicability. To overcome these limitations, future research should focus on AI‐driven frameworks capable of dynamically modeling and optimizing diverse TENG designs. For instance, DNNs can analyze complex relationships between variables such as layer thickness, surface roughness, and interlayer spacing, enabling data‐driven optimization across various TENG architectures. A DNN could identify material and structural combinations that maximize energy output and longevity under different operational conditions. RL offers additional potential, enabling TENG systems to autonomously adapt to fluctuating environmental conditions. By leveraging RL, TENGs could adjust parameters, such as contact frequency, layer alignment, and material compression in real time based on environmental feedback. This adaptability is particularly beneficial for applications with variable energy demands, like wearable electronics or autonomous IoT networks. AI‐based optimization has demonstrated potential performance enhancements of 30%–50% in simulations of adaptive TENG designs, with improvements observed in energy output and stability even under challenging conditions like high humidity or temperature variability.

### 4.3 Enhancing Data Accuracy and Measurement

In recent AI‐driven TENG research, a strong focus has been placed on enhancing energy output, predicting performance, and refining device parameters. Yet, a major unresolved challenge is achieving high data accuracy and consistency in TENG output measurements. Variability in measurements particularly in parameters like applied pressure, contact frequency, and electrical output leads to data inconsistencies that make it difficult to replicate results and compare findings across different studies. This lack of measurement standardization is a notable gap in TENG research, limiting the broader applicability and reliability of findings. To tackle these issues, implementing AI‐enabled universal testing machines presents a promising solution. These advanced testing systems could dynamically adjust for subtle variations in applied pressure and contact frequency during TENG operation, thereby reducing measurement errors and ensuring more consistent and reliable data. By automating these adjustments, AI‐driven testing machines would significantly improve measurement precision, even in experiments involving fluctuating environmental conditions or variable mechanical inputs, common in TENG applications. Moreover, integrating AI‐enhanced output measurement systems could standardize the way electrical outputs are recorded, addressing variations in voltage, current, and charge that often complicate result interpretation. These systems would ensure that electrical parameters are not only recorded accurately but also standardized across experiments, making data more comparable. For example, by applying ML algorithms capable of filtering noise and adjusting for minor inconsistencies in real‐time, these systems could provide cleaner, more precise measurements of TENG output characteristics. Improving measurement accuracy through AI also has broader implications for the reproducibility and robustness of TENG research. By establishing a standardized framework for evaluating TENG performance, researchers could more accurately compare findings across studies, leading to a more comprehensive understanding of TENG technology's capabilities and limitations. This standardization could help identify performance bottlenecks and guide the development of more reliable TENG devices for diverse applications. In practical terms, AI‐driven measurement accuracy improvements could result in performance assessments that are up to 20%–40% more reliable, based on initial tests using AI‐enabled calibration techniques.

### 4.4 Developing Universal Predictive Models for TENG Performance

While AI‐based models have shown significant potential in predicting TENG performance under specific conditions, they often lack generalizability, functioning effectively only within limited parameter ranges. For example, Jiang et al.^[^ ^29^ ^]^ utilized a DNN model to predict TENG performance with high accuracy, but the model was specifically calibrated for certain configurations, such as grating, disc, and rolling structures. While accurate within these parameters, such models struggle to extend their applicability to a broader array of TENG architectures and varied operational environments. This limitation restricts their utility in real‐world scenarios where conditions can vary widely. Advancing TENG predictive capabilities requires developing universal, adaptable AI models capable of accurately forecasting performance across diverse TENG designs and operational contexts. These models need sophisticated architectures to account for the complex, multivariable interactions in TENG systems, including material properties, environmental factors, structural designs, and operational conditions. Advanced AI approaches, such as ensemble NNs or generative models, could integrate diverse data inputs to produce more flexible and generalized predictive outcomes, enabling these models to apply across various TENG configurations. Incorporating reinforcement learning (RL) into predictive models could further enhance their adaptability by allowing dynamic responses to changing conditions. For instance, RL‐based TENG models could adjust to variations in environmental parameters such as temperature, humidity, and mechanical forces, enabling real‐time optimization of energy output, as discussed in Section 4.2. By learning continuously from feedback, these models could adapt to different operational states, ensuring stable performance even in fluctuating environments. The development of universal predictive models would mark a significant milestone in advancing TENG technology, broadening its applicability and making it more viable for diverse real‐world applications.

### 4.5 Expanding AI Algorithms for TENG Optimization

While traditional AI algorithms, such as DTs and SVMs, have been effective in specific TENG research applications, they often fall short in handling the complex, multidimensional data required for comprehensive TENG optimization. Emerging DL algorithms, including CNNs and RNNs, offer the potential for more advanced feature extraction and time‐series analysis, which are crucial for understanding and improving TENG performance across varying conditions. CNNs, known for their capacity to detect spatial patterns, could be particularly useful in identifying subtle structural variations in TENG layers. TENG efficiency depends heavily on factors like surface morphology, electrode design, and triboelectric layer structure. By applying CNNs to analyze high‐resolution imaging data of TENG surfaces, researchers can detect minute structural changes affecting performance. This enhances fault detection and quality control by identifying issues like material degradation or manufacturing inconsistencies early. CNN insights also support precise adjustments in surface texturing and layer composition, improving energy output and device longevity. RNNs, adept at processing sequential data, are well‐suited for analyzing time‐dependent variations in TENG output. Since TENGs operate under dynamic conditions with fluctuating variables such as applied force, contact frequency, and environmental factors, RNNs can interpret these temporal patterns for predictive maintenance. An RNN system could monitor output trends, forecast performance degradation, and recommend maintenance to minimize downtime and extend device life. Additionally, RNNs can enable real‐time adjustments to maximize efficiency based on historical data. RL offers a promising approach for real‐time optimization of TENG systems. RL operates through trial and error, enabling systems to learn optimal actions by interacting with their environment. RL algorithms can autonomously adjust parameters, such as electrode spacing, surface texture, or material composition in response to changes in temperature, humidity, or mechanical stress. For instance, an RL algorithm could adapt layer thickness or surface patterns in real time to sustain peak energy output under fluctuating conditions. RL‐driven optimization guided by historical data allows TENG systems to develop adaptive behaviors, increasing resilience to changing environments. Implementing RL systems would enable TENG devices to achieve higher operational efficiency with minimal human intervention, adapting dynamically to new conditions. This adaptability is particularly valuable for applications in dynamic or remote environments, such as wearable devices, environmental monitoring, or autonomous IoT networks.

### 4.6 Material Innovation for Sustainable TENG Development

Material selection is crucial in determining the efficiency, durability, and environmental impact of TENGs. While current TENG materials, including synthetic polymers like PTFE and PDMS, as well as nanomaterial‐enhanced layers, exhibit high performance, they often rely on nonrenewable resources and lack biodegradability raising concerns about their environmental footprint. Sustainable material innovation is therefore essential to align TENG technology with long‐term ecological goals, especially as demand for energy‐harvesting devices grows in fields, such as IoT and smart cities. Recent advances have leveraged AI in the discovery and optimization of triboelectric materials, focusing on synthetic compounds with excellent electron transfer capabilities. However, the field is now shifting toward identifying eco‐friendly, high‐performance alternatives. AI‐driven exploration of sustainable materials like cellulose‐based triboelectric layers or biodegradable composites could be transformative.^[^ ^208^ ^]^ Cellulose, for instance, is abundant, renewable, and has inherent triboelectric properties that can be enhanced for specific applications. By leveraging AI algorithms, researchers can efficiently analyze large datasets to predict material properties, such as electron affinity, dielectric constant, and mechanical resilience, identifying materials with optimal triboelectric properties that are also environmentally friendly. Beyond material identification, AI can simulate the effects of doping levels or nanomaterial additions on TENG performance. For example, nanofillers or nanoparticles functionalized in polymers can enhance charge density and durability. AI accelerates this process by modeling molecular interactions and structural modifications, enabling precise tuning of doping strategies for optimized performance. These insights allow for tailoring triboelectric properties to specific applications while upholding environmental sustainability. AI‐driven models for sustainable TENG materials align with global sustainability goals, supporting scalable, eco‐friendly infrastructure deployment.

### 4.7 Advancing TENG Standards through AI‐Powered Fabrication and Quality Control

A significant challenge in TENG research is the absence of standardized protocols for fabrication and testing, leading to inconsistencies in device performance and hindering the scalability and comparability of research findings. Variations in fabrication methods, from material selection to layer structuring and environmental conditions, can result in substantial performance discrepancies across devices. This lack of standardization not only complicates comparative research but also poses challenges for TENGs large‐scale deployment in applications that require reliability, such as biomedical devices, wearables, and IoT systems. Integrating AI into the fabrication and quality control process offers a promising solution to address these inconsistencies and establish reliable standards for TENG production. By embedding AI algorithms within fabrication machinery, production parameters, such as temperature, pressure, material deposition rates, and layer thickness can be monitored and adjusted in real‐time. This ensures consistent and reproducible output by automatically fine‐tuning conditions to meet predefined standards. For example, an NN model could analyze real‐time sensor data to detect deviations in layer uniformity or material alignment, adjusting fabrication settings immediately to correct variations. ML models further enhance quality control by detecting anomalies during production. Trained on extensive TENG production datasets, these systems can identify deviations from standard parameters that signal potential defects. For instance, ML algorithms could analyze data on surface morphology or electrical characteristics to recognize patterns indicating issues like poor layer adhesion or suboptimal charge retention. Predictive quality checks implemented via AI can address such problems immediately, reducing waste and ensuring only high‐quality devices move through the production line. Establishing a standardized, AI‐driven framework for TENG fabrication would be invaluable for industries requiring both high reliability and scalability.

### 4.8 Bridging the Gap in AI‐Powered TENG Measurement and Reporting

For AI‐driven advancements in TENG technology to achieve their full potential, standardized measurement and reporting practices are essential. Currently, inconsistencies in data capture, processing, and reporting create significant barriers to comparing and validating findings across studies, limiting reproducibility and scalability. Implementing AI‐based measurement and reporting systems can establish a universal framework for data collection and analysis, thereby enhancing the reliability and comparability of TENG performance metrics. AI‐enabled reporting systems can automatically record, analyze, and standardize TENG output data, reducing human error and ensuring consistency across studies. For instance, an AI‐driven electrical measurement system could dynamically adapt to varying TENG output ranges, accurately capturing voltage, current, and charge data while standardizing output formats. Such adaptability is crucial because TENG devices often exhibit variable performance depending on design, materials, and environmental conditions. By intelligently calibrating to these variations, AI systems ensure that each data point is directly comparable and that key metrics, such as efficiency, power density, and longevity, are consistently recorded.

Additionally, AI can facilitate real‐time data validation by cross‐referencing measurements against expected parameters or historical benchmarks, instantly flagging outliers or inconsistencies that may indicate experimental errors or device anomalies. This capability enhances data quality and reliability, providing researchers with a robust foundation for further analysis. Automating data processing and reporting also accelerates research workflows, allowing scientists to focus more on interpretation and application rather than manual data handling. Standardized measurement and reporting practices supported by AI are equally critical for validating AI‐driven predictive models across diverse research contexts and applications. A cohesive, universally accepted data framework would enable researchers globally to contribute to and utilize a shared data repository, significantly accelerating innovation in TENG technology. High‐quality, consistent data are essential for training AI models accurately, particularly for applications where TENGs are deployed under varying environmental and operational conditions, such as biomedical devices, remote sensing systems, and smart infrastructure. By ensuring uniformity and precision in data collection, AI‐driven standardization can address fundamental challenges in TENG research, paving the way for scalable and reproducible advancements across diverse industries.

### 4.9 Creating Low‐Power AI Algorithms for Self‐Sustaining TENG Systems

A critical future direction for advancing TENG applications lies in the development of low‐power AI algorithms specifically tailored to these energy‐constrained systems as discussed in Section 4.1. Since TENGs generate limited energy, it is essential that any AI algorithms they employ are optimized to function within these constraints. Traditional AI models often require significant computational resources, which could drain the limited energy produced by TENGs, undermining the self‐sustaining advantage these devices offer. Therefore, the focus is on creating power‐efficient AI algorithms that can operate effectively without depleting the energy TENGs harvest. To achieve this, researchers are exploring lightweight AI approaches, such as edge AI and neuromorphic computing techniques, which offer significant reductions in power consumption. Edge AI enables data processing on the device itself, reducing the need for power‐intensive data transmission to external servers. In TENG systems, edge AI algorithms can perform real‐time analysis and decision‐making, directly on the device, allowing for efficient management of power resources. Neuromorphic computing represents another promising approach, using architectures inspired by the human brain to mimic neural pathways, which inherently require less energy for processing tasks compared to conventional digital models. Neuromorphic processors could allow TENG‐powered devices to perform complex tasks, like pattern recognition or anomaly detection while consuming minimal power. This computing style not only aligns well with the low‐power nature of TENGs but also provides the adaptability necessary for real‐time operation in dynamic environments. By minimizing computational load, these power‐efficient algorithms could support sustainable TENG operation in applications where other power solutions are unfeasible.

### 4.10 Scaling AI‐Optimized TENGs for Mass Production

The large‐scale deployment of AI‐enhanced TENGs presents significant challenges, primarily due to fabrication variability, material costs, and performance consistency. The absence of standardized manufacturing protocols leads to inconsistencies in material properties, triboelectric charge retention, and surface morphology, which directly affect energy conversion efficiency. To address these issues, AI‐integrated fabrication techniques leveraging real‐time monitoring and ML‐based anomaly detection can enhance production uniformity. By employing AI‐driven predictive quality control, defects can be detected at early stages, reducing material waste and optimizing manufacturing yield. Furthermore, AI‐guided self‐calibration mechanisms dynamically adjust fabrication parameters, compensating for process‐induced variations and ensuring batch‐to‐batch consistency in performance. Another major constraint in scaling AI‐TENGs is the economic feasibility of high‐performance triboelectric materials. While nanocomposites and bio‐inspired coatings exhibit superior energy‐harvesting efficiency, their large‐scale production remains cost‐prohibitive. AI‐driven material discovery frameworks facilitate the identification of cost‐effective alternatives with comparable triboelectric properties, optimizing material selection for large‐scale applications. AI‐assisted predictive modeling enables the refinement of structural parameters before fabrication, minimizing experimental iterations and material wastage. Moreover, the integration of AI with scalable manufacturing methodologies, such as roll‐to‐roll processing, inkjet printing, and additive manufacturing, can significantly reduce production costs while ensuring high device reliability and structural integrity. For AI‐TENGs to be viable in large‐scale applications such as IoT‐based sensor networks, smart infrastructure, and self‐powered electronic systems, their performance must remain stable under varying environmental conditions. External factors, including fluctuations in humidity, temperature, and mechanical loading, influence triboelectric charge generation, leading to potential performance degradation. AI‐driven self‐adaptive control mechanisms enable real‐time adjustments in electrode configurations, surface modifications, and charge transfer pathways to optimize energy harvesting efficiency under dynamic operational conditions. Additionally, edge AI and federated learning approaches facilitate decentralized processing, reducing computational latency and enhancing system autonomy while minimizing the dependency on energy‐intensive cloud computing infrastructures. The successful integration of AI into TENG mass production necessitates a multidisciplinary approach combining advanced fabrication techniques, cost‐efficient material innovations, real‐time adaptive control, and energy‐efficient AI models

### 4.11 Establishing Robust Security Protocols for AI‐Enhanced TENG Applications

As AI‐enhanced TENGs are increasingly integrated into critical domains such as healthcare, smart cities, and industrial automation, ensuring robust security protocols is essential to protect sensitive data and maintain system integrity. These AI‐driven TENG systems continuously collect, process, and transmit information, making them susceptible to cyber threats, unauthorized access, and data breaches. Without stringent security measures, these vulnerabilities could compromise both user privacy and device functionality, particularly in applications, such as medical monitoring and real‐time industrial control. Therefore, developing security frameworks tailored to the constraints and capabilities of TENG‐powered applications is a priority for future research. A key approach to enhancing security in AI‐TENG systems is federated learning, which enables decentralized AI training by keeping data on local devices while only sharing anonymized model updates with a central system. This technique significantly reduces exposure to external threats, making it particularly suitable for privacy‐sensitive applications, such as healthcare. For instance, AI‐powered TENG wearables can process patient health data directly on the device, minimizing the risk of transmitting sensitive information over potentially insecure networks. Federated learning ensures that AI‐driven insights can be generated while preserving data confidentiality, making it an optimal solution for secure, self‐powered AI‐TENG systems. In addition to federated learning, lightweight encryption protocols designed for energy‐constrained TENGs are crucial for securing data transmission in connected environments. These encryption techniques protect information exchanged between TENG‐powered sensors and larger networks, preventing interception and unauthorized access. This is particularly critical in applications, such as real‐time traffic monitoring and industrial automation, where data integrity directly influences decision‐making processes. By incorporating optimized cryptographic algorithms, AI‐TENG systems can maintain secure communications without imposing excessive computational burdens on the energy‐harvesting devices. Furthermore, ML‐based anomaly detection plays a vital role in identifying and mitigating security threats in real time. AI models can continuously monitor device behavior, detecting deviations that may indicate cyber intrusions or unauthorized manipulations. To further enhance data protection, integrating secure multiparty computation (SMPC) and homomorphic encryption can enable encrypted data analysis without exposing underlying information, thereby strengthening privacy in AI‐TENG deployments. As AI‐TENG technology scales for broader adoption, establishing standardized cryptographic frameworks, edge AI privacy solutions, and adaptive security architectures will be essential for ensuring their secure and reliable implementation across diverse sectors. These security advancements will not only safeguard sensitive data but also support the widespread adoption of AI‐enhanced TENGs in critical, self‐powered IoT and medical applications.

## 5 Future Research Directions and Data Insights for Advancing AI‐Driven TENGs

To advance AI‐enhanced TENG technology, future research should target several critical areas outlined in Sections 4.1–4.10. Each area addresses specific challenges while paving the way for new applications in IoT, healthcare, and smart city infrastructure. Key research directions include:

1. Low‐Power AI Algorithm Development: Designing ultralow‐power AI algorithms, such as TinyML, tailored for TENG‐powered systems to enable real‐time, local data processing with minimal energy consumption. These algorithms support autonomous operations in remote or inaccessible areas, such as monitoring patient vitals or environmental conditions, without depleting energy reserves.
2. Flexible and Biocompatible Material Innovation: Advancing biodegradable and flexible materials like PLA, graphene, and silk fibroin to create environmentally friendly and biocompatible TENGs. These materials enable integration into implants and wearables, addressing the need for comfort, flexibility, and sustainability in biomedical and health monitoring applications.
3. Hybrid Energy Systems for Continuous Power Supply: Combining TENGs with complementary energy sources, such as photovoltaics or miniaturized fuel cells, to overcome intermittent power limitations. Hybrid systems capable of generating up to 200 Wh per day would ensure uninterrupted power for remote IoT devices and critical off‐grid applications.
4. Advanced Triboelectric Material Engineering: Exploring nanoscale surface modifications and advanced composites with high electron affinity to boost energy conversion efficiency by up to 40%. Enhanced materials will make TENGs more viable for large‐scale energy harvesting and high‐demand applications.
5. Secure Data Protocols: Establishing robust security measures, including lightweight encryption and blockchain technologies, to protect sensitive data in IoT and healthcare applications. Federated learning can further enhance privacy by keeping data local, while encryption ensures secure data transmission across networks.
6. Adaptive AI Interfaces for Personalized Applications: Developing adaptive AI systems that personalize energy generation based on user behavior or environmental conditions. By analyzing activity patterns, AI‐driven TENGs can optimize parameters to improve energy availability for wearables and other consumer‐centric devices.
7. Energy‐Efficient Manufacturing and Scalability: Leveraging AI for precision manufacturing of TENG devices to ensure scalability and standardization. This approach supports cost‐effective production while maintaining high‐quality standards.
8. Optimization of Material Properties: Certain materials such as textile fabrics for example are non‐linear and their viscoelastic behavior can cause signal distortion leading in not inconsistent energy output and signal distortion. Knowledge of their properties under dynamic conditions that involve combined forces [223] needs ML to normalize them.
9. Universal Predictive Models for TENG Optimization: Creating adaptable AI models that accurately predict TENG performance across diverse configurations and conditions, ensuring consistent and reliable functionality in various applications.
10. Real‐Time Performance and Anomaly Detection: Employing Edge AI strategies to enable local, energy‐efficient processing for real‐time adaptability, anomaly detection, and predictive maintenance in TENG‐powered systems, particularly for IoT and industrial automation.
11. Interdisciplinary Collaboration: Encouraging partnerships across materials science, AI, and engineering to accelerate innovation, focusing on sustainability, efficiency, and application‐specific optimization.

These areas collectively address energy efficiency, security, material sustainability, and personalization, unlocking the full potential of AI‐enhanced TENGs for intelligent, self‐powered devices in diverse industries.

## 6 Conclusion

TENGs hold great promise as self‐powered sensors and auxiliary battery chargers, offering the potential to reduce reliance on traditional power sources and extend the operational lifespan of electronic devices. However, achieving this vision requires overcoming significant challenges in material selection, synthesis, fabrication, and design optimization. The integration of AI with TENG systems presents a transformative pathway to address these complexities by leveraging AI's capabilities in data analysis, pattern recognition, and adaptability. ML and DL techniques enable TENG systems to dynamically adapt to changing environments, fostering intelligent, self‐powered devices capable of real‐time monitoring and autonomous adjustment. This review highlights how AI‐driven methodologies can enhance every phase of TENG development, from material selection to fabrication and application deployment. AI improves TENG performance by optimizing energy harvesting efficiency, enabling predictive maintenance, and fine‐tuning performance under dynamic conditions. ML and DL models facilitate the development of TENG systems with superior configurations, optimized material choices, and higher energy conversion efficiencies, advancing their practicality and cost‐effectiveness. Despite these advancements, challenges remain. Critical concerns include data privacy, scalability, sustainable material sourcing, and energy storage solutions. In sensitive applications, such as healthcare, data privacy demands robust security measures. Scalability is essential for broader implementation in urban infrastructure and industrial settings, while sustainable materials are crucial to aligning TENG technology with eco‐friendly goals. Future research directions include the development of low‐power AI algorithms for energy‐efficient processing, the use of biodegradable materials to minimize environmental impact, the design of hybrid energy systems for stable power supply, and the establishment of stringent data security protocols. The convergence of AI and TENG technologies will require collaborative efforts across materials science, AI, and engineering disciplines to create adaptive, self‐sustaining devices. This review underscores the potential of AI‐enhanced TENGs as foundational technologies for sustainable innovation, advancing energy independence and promoting eco‐friendly solutions for a rapidly evolving world

## Conflict of Interest

The authors declare no conflict of interest.
